# The epidemiology of hepatitis C virus in Pakistan: systematic review and meta-analyses

**DOI:** 10.1098/rsos.180257

**Published:** 2018-04-11

**Authors:** Zaina Al Kanaani, Sarwat Mahmud, Silva P. Kouyoumjian, Laith J. Abu-Raddad

**Affiliations:** 1 Infectious Disease Epidemiology Group, Weill Cornell Medicine-Qatar, Qatar Foundation - Education City, PO Box 24144, Doha, Qatar; 2 Department of Healthcare Policy and Research, Weill Cornell Medicine, Cornell University, New York, NY, USA

**Keywords:** Hepatitis C virus, epidemiology, prevalence, incidence, Middle East and North Africa

## Abstract

To characterize hepatitis C virus (HCV) epidemiology in Pakistan and estimate the pooled mean HCV antibody prevalence in different risk populations, we systematically reviewed all available records of HCV incidence and/or prevalence from 1989 to 2016, as informed by the Cochrane Collaboration Handbook. This systematic review was reported following the PRISMA guidelines. Populations were classified into six categories based on the risk of exposure to HCV infection. Meta-analyses were performed using DerSimonian and Laird random-effects models with inverse variance weighting. The search identified one HCV incidence study and 341 prevalence measures/strata. Meta-analyses estimated the pooled mean HCV prevalence at 6.2% among the general population, 34.5% among high-risk clinical populations, 12.8% among populations at intermediate risk, 16.9% among special clinical populations, 55.9% among populations with liver-related conditions and 53.6% among people who inject drugs. Most reported risk factors in analytical epidemiologic studies related to healthcare procedures. Pakistan is enduring an HCV epidemic of historical proportions—one in every 20 Pakistanis is infected. HCV plays a major role in liver disease burden in this country, and HCV prevalence is high in all-risk populations. Most transmission appears to be driven by healthcare procedures. HCV treatment and prevention must become a national priority.

## 1. Introduction

Hepatitis C virus (HCV) is a blood-borne pathogen and a significant global health concern [1]. Following the acquisition of the virus, acute HCV infection can progress to chronic infection [2], which is associated with several morbidities, such as liver cirrhosis and cancer [3–5]. HCV-related morbidity strains healthcare systems worldwide, with approximately 71 million people chronically infected globally [6]. Direct-acting antivirals (DAAs), a highly efficacious HCV treatment, can clear HCV infection and may substantially reduce HCV disease burden and onward transmission [7]. As such, global targets have been set by the World Health Organization (WHO) to eliminate HCV infection by 2030 [8,9].

The Middle East and North Africa (MENA) region is the most affected region by HCV infection, with approximately 15 million individuals chronically infected [6]. HCV is highly endemic in Pakistan, where a national survey, conducted in 2007–2008, estimated HCV prevalence at 4.8% [10]. Ongoing transmission appears to be widespread, occurring in both healthcare and community settings [10]. Understanding HCV epidemiology in Pakistan is critical in developing and targeting cost-effective prevention and treatment interventions against HCV, in order to meet the global target of HCV elimination.

The objective of this systematic review is to characterize HCV epidemiology in Pakistan by: (i) systematically reviewing and synthesizing available published data of HCV incidence and prevalence in six population categories defined according to risk of exposure and (ii) pooling available HCV prevalence measures in each of the six pre-defined risk population categories to estimate population-specific pooled mean HCV prevalence.

This work was conducted as part of the MENA HCV Epidemiology Synthesis Project, which aims to characterize HCV epidemiology in MENA to inform key public health research, policy, programming and resource allocation priorities [11–24].

## 2. Methods

The methodology used in this study follows that used in previous systematic reviews of the MENA HCV Epidemiology Synthesis Project [11–17]. The subsequent subsections summarize this methodology. Further details are available in previous publications [11–17].

### 2.1. Data sources and search strategy

All available records reporting HCV incidence and/or prevalence measures in Pakistan were systematically reviewed, as informed by the Cochrane Collaboration Handbook [25]. Results were reported using the Preferred Reporting Items for Systematic and Meta-analyses (PRISMA) guidelines (electronic supplementary material, table S1) [26]. Our main data sources included PubMed and Embase databases. Broad search criteria (electronic supplementary material, figure S1) were used to retrieve articles and abstracts on PubMed and Embase, from 1989 (the year in which HCV was first identified [27,28]) up to 19 April 2016, with no language restrictions.

### 2.2. Study selection

**Figure 1.**
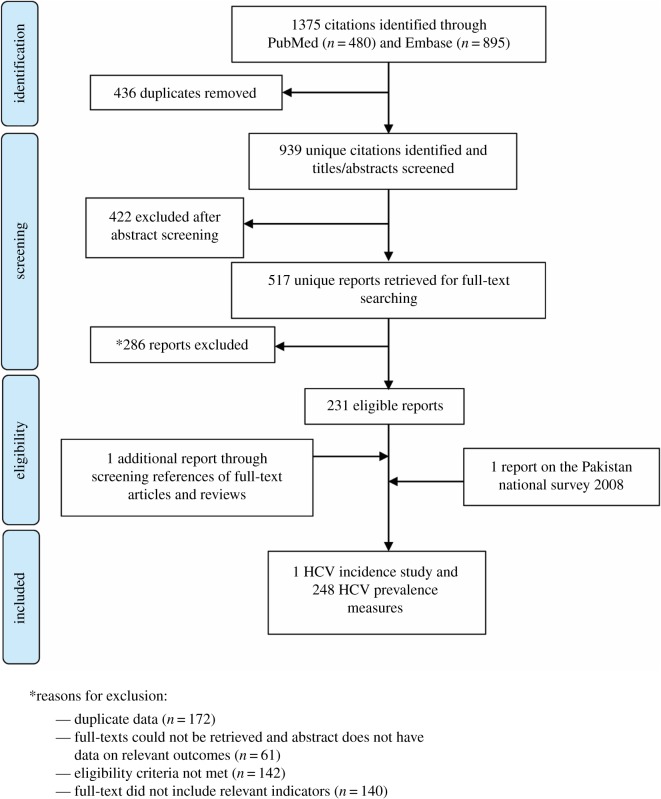
Flow chart of article selection for the systematic review of hepatitis C virus (HCV) incidence and prevalence in Pakistan, adapted from the PRISMA 2009 guideline [26].

Similar to our previous systematic reviews [11–17], all records identified through our search were imported into the reference manager Endnote, where duplicate publications were identified and excluded. The remaining unique reports were subjected to a two-stage screening process, performed by Z.A.K. and S.P.K. In the first stage, titles and abstracts were screened for relevance. Records marked as relevant or potentially relevant proceeded to the second stage of screening, in which full-texts were obtained and assessed for eligibility based on predetermined inclusion/exclusion criteria. Eligible reports were included in this study, and ineligible reports were excluded with reasons specified in figure 1. Additional records were identified by screening references in full-text articles and the literature reviews, as well as a country-level report.

### 2.3. Inclusion and exclusion criteria

The inclusion and exclusion criteria used in this study were adapted from our previous systematic reviews [11–17]. Briefly, any article reporting HCV antibody incidence and/or antibody prevalence, based on primary data, qualified for inclusion in this review. An article was excluded if it was a case report, case series, editorial, letter to editor(s), commentary, review, referred to HCV as non-A non-B hepatitis, contained duplicate information, reported HCV prevalence based on self-reporting, and if the study population was Pakistani nationals residing outside Pakistan.

In this work, for clarity, a ‘report’ refers to a document (article, conference abstract, country-level report and others) including one or several outcome measures of those included in our systematic review, while a ‘study’ refers to any one specific single outcome measure. One report may contribute multiple studies (say several prevalence measures in different populations), and multiple reports of the same outcome measure (say same prevalence measure in the same specific sample) were identified as duplicates and deemed as one study.

### 2.4. Data extraction and data synthesis

Data from relevant reports were extracted by Z.A.K., of which 20% were double extracted by S.P.K. to ensure consistency. Nature of extracted data followed our previous systematic reviews [11–17]. HCV prevalence measures were extracted and reported as per original reports. These measures were rounded to one decimal place except for measures below 0.1%, which were rounded to two decimal places.

Risk factors that were found to be significantly associated with HCV infection through multivariable regression analyses were extracted. HCV ribonucleic acid (RNA) prevalence among HCV antibody-positive individuals (that is HCV viraemic rate [20]) was extracted whenever available in reports including an HCV prevalence.

The extracted data were synthesized by risk population in six distinct categories defined according to the risk of exposure to HCV infection as follows:

1. General population (populations at low risk): these included blood donors, pregnant women, children, refugees, household-based survey participants and national army recruits, among others.
2. High-risk clinical populations: these included populations exposed to frequent medical injections and/or blood transfusions, such as haemodialysis, thalassaemia, haemophilia and multi-transfused patients, among others.
3. Populations at intermediate risk: these included populations whose risk of exposure is higher than the general population but lower than populations at high risk, such as healthcare workers (HCWs), household contacts of HCV-infected patients, patients with diabetes and prisoners, among others.
4. Special clinical populations: these included clinical populations whose risk of exposure to HCV infection is difficult to ascertain, such as patients with non-liver-related malignancies, dermatological manifestations and rheumatological disorders, among others.
5. Populations with liver-related conditions: these included patients with liver-related conditions of an epidemiological significance to HCV infection such as patients with chronic liver disease, acute viral hepatitis, hepatocellular carcinoma and liver cirrhosis, among others.
6. People who inject drugs (PWID).

### 2.5. Quantitative analysis

The quantitative analysis approach was similar to that in our previous HCV systematic reviews [11–17]. HCV prevalence measures were presented by risk population in reports with a sample size greater than or equal to 50 in tables 1–3; electronic supplementary material, S2–S4. If no explicit HCV prevalence measure was reported, it was calculated based on the sample size and number of events reported, if available. HCV prevalence for the total sample was replaced with stratified measures, whenever the sample size was greater than or equal to 25 participants for each stratum. Stratified data were included using a pre-defined order that prioritizes stratifications by population followed by sex, year, region and age. Meta-analyses were conducted for studies/strata with a minimum sample size of 25 participants. Only one final stratification per study was included in the meta-analyses.

**Table 1.** Studies reporting hepatitis C virus (HCV) prevalence among the general population (populations at low risk) in Pakistan. Prev, prevalence; CC, case-control; CS, cross-sectional; Conv, convenience; MsRS, multi-stage random sampling, RCS, random cluster sampling; SRS, simple random sampling; SsCS, single-stage cluster sampling; NWFP, North West Frontier Province; NHL, non-Hodgkin's lymphoma.

author (citation)	year(s) of data collection	province or city	study site	study design	study sampling procedure	population	sample size^a^	HCV prev^b^ (%)
Agboatwalla [29]	1990–1991	—	community	CS	Conv	healthy children	226	0.4
Kakepoto [30]	1989–1994	Karachi and Hyderabad	blood donation camps	CS	Conv	blood donors	16 705	1.2
Luby [31]	1993	Hafizabad	community	CS	RCS	general population	309	6.5
Parker [32]	—	Lahore	hospital	CS	Conv	pregnant women	417	6.7
Parker [32]	—	Lahore	hospital	CS	Conv	children	538	1.3
Mujeeb [33]	1996–1997	Karachi	medical centre	CS	Conv	blood donors from students' community	612	0.5
Khan [34]	1995	Darsano Channo Karachi	general clinic	CS	Conv	outpatients from health clinics	135	44.0
Mujeeb [35]	1997–1998	Karachi	medical centre	CS	Conv	replacement blood donors	7047	2.4
Aslam [36]	2000	Lahore	community	CS	Conv	general population	488	15.9
Aslam [36]	2000	Gujranwala	community	CS	Conv	general population	1922	23.8
Khattak [37]	1996–2000	Rawalpindi	community	CS	Conv	healthy blood donors	103 858	4.1
Qureshi [38]	1996–1999	Karachi	blood bank in a hospital	CS	Conv	blood donors	401	4.5
Mumtaz [39]	2001–2002	Rawalpindi	hospital	CS	Conv	healthy blood donors	563	6.2
Asif [40]	2002–2003	Northern Pakistan	blood transfusion unit	CS	Conv	replacement blood donors	3187	5.1
Asif [40]	2002–2003	Northern Pakistan	blood transfusion unit	CS	Conv	voluntary blood donors	243	2.5
Khokhar [41]	2001–2002	Islamabad	hospital	CS	Conv	pregnant women	503	4.8
Aslam [42]	—	Lahore	community	CS	Conv	general population	523	14.9
Jaffery [43]	2001–2002	Islamabad	hospital	CC	Conv	pregnant women	947	3.3
Muhammad [44]	1998–2002	Buner, NWFP	hospital	CS	Conv	outpatients	16 400	4.6
Jafri [45]	2003–2004	Karachi	community	CS	Conv	healthy children	3533	1.6
Mujeeb [46]	2000	Karachi	medical centre	CS	Conv	first time replacement blood donors	7325	3.6
Rifat-uz [47]	2004	Bahawalpur	community	CS	Conv	general population	6815	4.4
Ahmad [48]	2004	Faisalabad	hospital	CS	SRS	blood donors and general population	300	20.6
Bhatti [49]	2003–2005	Rawalpindi	blood transfusion unit	CS	Conv	blood donors	94 177	4.2
Bhatti [49]	2004	Rawalpindi	blood transfusion unit	CS	Conv	blood donors	966	3.8
Sultan [50]	1996–2005	Lahore	tertiary care centre	CS	Conv	replacement blood donors	41 498	3.7
Abbas [51]	—	Sukkar	community	CS	SCS	general population	873	33.7
Butt [52]	2004–2005	—	hospital	CS	Conv	army recruits	5707	1.7
Hakim [53]	2002–2006	Karachi	university	CS	Conv	female university students	4000	5.2
Idrees [54]	1999–2007	Punjab	community	CS	Conv	general population	6817	15.1
Khattak [55]	—	Peshawar	blood banks in hospitals	CS	Conv	male blood donors	1131	4.1
Mujeeb [56]	2004–2007	Sindh	blood bank in a medical centre	CS	Conv	blood donors	5345	7.5
Abbas [57]	2005–2008	—	liver clinic	CS	Conv	blood donors	804	14.0
Ali [58]	2003–2005	Multan	community	CS	SRS	general population	116	6.7
Bangash [59]	2007	Parachinar and Sadda	blood transfusion unit	CS	Conv	blood donors	1300	1.6
Bangash [60]	2007–2008	Parachinar	blood bank in a hospital	CS	Conv	blood donors	10 343	0.4
Gul [61]	2006–2007	Abbottabad	hospital	CS	Conv	pregnant women	500	8.9
Jalbani [62]	—	Khairpur Nathan Shah and Shahdadkot	community	CS	Conv	general population	406	30.3
Junejo [63]	2007–2008	Hyderabad	hospital	CS	Conv	outpatients	931	17.2
Hussain [64]	2008	Karachi	blood bank	CS	Conv	blood donors	98 012	6.0
Sami [65]	2005	Karachi	medical centre	CS	Conv	pregnant women	5902	1.8
Shaikh [66]	2006–2007	Larkana city	general clinic	CS	Conv	general population	450	6.6
Sheikh [67]	2006	Karachi	Hospital	CS	Conv	pregnant women	2592	0.7
Abbas [68]	—	Karachi	community	CS	Conv	general population	504	3.2
Ali [69]	2009–2010	Khyber Pakhtunkhwa	community	CS	SRS	healthy inhabitants of District Mansehra	400	7.0
Aziz [70]	2007–2008	Sindh	community	CS	Conv	general population from peri-urban area	129	3.9
Aziz [70]	2007–2008	Sindh	community	CS	Conv	general population from rural area	388	28.6
Hashmi [71]	2006	Islamabad	community	CS	MsRS	female inhabitants: 15–50 years old	252	24.6
Hyder [72]	2007–2009	Punjab	community	CS	Conv	healthy men: 16–59 years old	58 680	6.9
Jadoon [73]	2008	—	blood bank in a hospital	CS	Conv	healthy blood donors	550	8.2
Jadoon [74]	—	Multan	hospital	CC	Conv	blood donors	10 000	4.9
Jamil [75]	2010	Tehsil Oghi	community	CS	Conv	general population	648	10.3
Janjua [76]	2005	Karachi	community	CS	SCS	general population	1997	23.8
Qureshi [10]	2007–2008	All regions of Pakistan	national	CS	MsRS	household survey members	47 043	4.8
Shah [77]	2007–2008	Karachi	hospital	CS	Conv	blood donors	32 042	1.6
Taseer [78]	2006–2007	Multan	hospital	CS	Conv	pregnant women	500	7.0
Aziz [79]	2005–2009	Karachi	hospital	CS	Conv	pregnant women: 18–45 years old	18 000	5.8
Borhany [80]	2007–2009	Karachi	specialized clinic	CS	Conv	blood donors	5717	1.9
Iqbal [81]	—	Gadap Town, Karachi	community	CS	Conv	previously unscreened adults: more than 10 years old	600	5.0
Khan [82]	2009	Khyber Pakhtunkhwa	blood bank	CS	Conv	voluntary blood donors	7148	1.9
Rauf [83]	2009	NWFP	refugee camp	CS	Conv	refugees in Baghicha Dheri camps	590	8.8
Safi [84]	2008–2009	NWFP	blood bank in a hospital	CS	Conv	blood donors	62 251	2.6
Saleem [85]	2008	Azad Kashmir	hospital	CS	Conv	outpatients	9564	6.4
Yousaf [86]	—	all regions of Pakistan	laboratory	CS	SRS	general population	120	29.2
Ahmed [87]	2007–2009	Balochistan	community	CS	MsRS	general population	2000	5.5
Ansari [88]	2010	Karachi	specialized clinic	CS	Conv	blood donors	5517	1.9
Attaullah [89]	2008–2011	Khyber Pakhtunkhwa	blood bank in a hospital	CS	Conv	blood donors	127 828	2.5
Bhutta [90]	2010	Sargodha	hospital	CS	Conv	replacement blood donors	100	12.0
Hafeez-ud [91]	2010	Punjab	community	CS	Conv	healthy adult males	14 027	3.1
Ijaz [92]	2011–2012	Lahore	hospital	CS	Conv	blood donors	3652	12.4
Khan [93]	2008	Lakki Marwat	hospital	CS	Conv	outpatients	1443	4.4
Khan [94]	2009–2012	Peshawar	hospital	CS	Conv	blood donors	6513	1.1
Memon [95]	2007–2008	Karachi	private security company	CS	Conv	security personnel	457	9.0
Muhammad [96]	2009–2010	Sindh	medical centre	CC	Conv	family members of NHL patients (controls)	584	7.7
Nawaz [97]	2011	—	hospital	CS	Conv	general population	435	12.2
Waheed [98]	2010	Islamabad	hospital	CS	Conv	blood donors	10 145	8.3
Abbas [99]	—	Balochistan	community	CS	Conv	general population	2800	7.0
Butt [100]	2013	Lahore	blood bank in a hospital	CS	Conv	male blood donors	833	1.9
Irfan [101]	2004–2010	Karachi	hospital	CS	Conv	blood donors	108 598	2.7
Khan [102]	2011	Quetta, Balochistan	hospital	CS	Conv	male blood donors	356	20.8
Khan [103]	2010–2011	Karachi	community	CS	SRS	household survey members	679	8.0
Qadeer [104]	2007–2012	Punjab	community	CS	Conv	blood donors from students’ community	5000	4.1
Rauf [105]	2011	Karachi	community	CS	Conv	male garbage scavengers	117	8.5
Seema [106]	2010	Hyderabad Sindh	hospital	CS	Conv	pregnant women	3078	4.7
Zaffar [107]	—	—	transfusion unit	CS	Conv	blood donors	246 611	2.9
Ali [108]	—	Mardan	hospitals and clinics	CS	Conv	general population	1419	11.7
Ilyas [109]	2013–2014	Peshawar	community	CS	Conv	general population	982	13.4
Moiz [110]	2011–2012	Southern Pakistan	hospital	CS	Conv	healthy noncommercial blood donors	42 830	1.7
Parveen [111]	2013	Multan	hospital	CS	Conv	potential employees sent for HCV screening	10 666	2.9
Kumari [112]	2012	Karachi	hospital	CS	Conv	pregnant women	300	13.3
Niazi [113]	2012–2013	Rawalpindi	transfusion unit	CS	Conv	blood donors	56 772	1.8
Sheikh [114]	—	Gwadar Port	disaster management camp	CS	Conv	blood donors in rural areas	300	4.3
Donchuk [115]	2015–2016	Karachi	community	CS	Conv	outpatients	4589	27.0
Karim [116]	2015	Mardan	hospitals	CS	Conv	blood donors	5318	1.1

^a^The table reports only studies whose sample size is greater than or equal to 50 participants. For space considerations, the table shows the overall HCV measure of each study rather than stratifications within population subgroups.

^b^The decimal places of the prevalence figures are as reported in the original report, but prevalence figures with more than one decimal place were rounded to one decimal place, with the exception of those below 0.1%.

**Table 2.** Studies reporting hepatitis C virus (HCV) prevalence among high-risk clinical populations in Pakistan.

author (citation)	year(s) of data collection	province or city	study site	study design	study sampling procedure	population	sample size^a^	HCV prev^b^ (%)
Mujeeb [117]	—	Karachi	medical centre	CS	Conv	thalassaemia patients	91	50.5
Gul [118]	1999	Lahore	haemodialysis unit	CC	Conv	haemodialysis patients	50	68.0
Khokhar [119]	2002–2003	Islamabad	hospital	CS	Conv	haemodialysis patients	97	23.7
Mumtaz [120]	2008	Lahore	hospital	CS	Conv	haemodialysis male patients	50	28.0
Ullah [121]	—	Karachi	hospital	CS	Conv	thalassaemia patients	79	43.0
Khan [122]	2010	Khyber Pakhtunkhwa	hospitals	CS	SRS	haemodialysis patients	384	29.2
Borhany [80]	2007–2009	Karachi	specialized clinic	CS	Conv	haemophilia patients	173	51.4
Riaz [123]	2009	Karachi	hospital	CS	Conv	thalassaemia patients (multi-transfused)	79	45.5
Ansari [88]	2010	Karachi	specialized clinic	CS	Conv	thalassaemia patients	160	13.1
Sadiq [124]	2008–2009	Lahore	hospital	CS	Conv	transfusion dependent children	120	54.2
Daud [125]	2008–2012	Islamabad	HIV care centre	CS	Conv	HIV patients who use drugs (non-intravenously)	81	6.2
Din [126]	2013	Rawalpindi	transfusion unit	CS	Conv	thalassaemia patients	95	49.5
Mahmud [127]	2012–2013	Karachi	—	CS	Conv	haemodialysis patients	189	16.4
Chishti [128]	2010–2011	Karachi	medical centre	CS	Conv	haemodialysis patients (multi-transfused)	200	29.0
Khan [129]	2013–2014	Khyber Pakhtunkhwa	hospitals	CS	Conv	thalassaemia patients	180	7.8
Yasmeen [130]	2012–2013	—	hospital	CS	Conv	thalassaemia patients	300	47.3

^a^The table reports only studies whose sample size is greater than or equal to 50 participants. For space considerations, the table shows the overall HCV measure of each study rather than stratifications within population subgroups.

^b^The decimal places of the prevalence figures are as reported in the original report, but prevalence figures with more than one decimal were rounded to one decimal place, with the exception of those below 0.1%. Prev, prevalence; CC, case-control; CS, cross-sectional; Conv, convenience; SRS, simple random sampling.

**Table 3.** Studies reporting hepatitis C virus prevalence among people who inject drugs (PWID) in Pakistan.

author (citation)	year(s) of data collection	province or city	study site	study design	study sampling procedure	population	sample size^a^	HCV prev^b^ (%)
Kuo [131]	2003	Lahore and Quetta	outpatient centres	CS	Conv	PWID	351	88.0
Achakzai [132]	2004	Quetta	community	CS	Conv	PWID	50	60.0
Altaf [133]	2003	Karachi	rehabilitation centre	CS	Conv	PWID	161	94.3
Abbasi [134]	2003	Quetta	community	CS	Conv	PWID	300	44.7
Platt [135]	2007	Rawalpindi	community	CS	RDS	PWID	302	17.3
Platt [135]	2007	Abbottabad	community	CS	RDS	PWID	102	8.0
Rehan [136]	2004	Karachi	community	CS	SRS	PWID	399	87.0
Rehan [136]	2004	Lahore	community	CS	SRS	PWID	380	91.8
Rehman [137]	—	Khyber Pakhtunkhwa	community	CS	Conv	PWID	200	31.5
Memon [95]	2007–2008	Karachi	laboratory	CS	Conv	PWID	407	68.3
Daud [125]	2008–2012	Islamabad	HIV care centre	CS	Conv	HIV patients who inject drugs	81	77.8

^a^The table reports only studies whose sample size is greater than or equal to 50 participants. For space considerations, the table shows the overall HCV measure of each study rather than stratifications within population subgroups.

^b^The decimal places of the prevalence figures are as reported in the original report, but prevalence figures with more than one decimal were rounded to one decimal place, with the exception of those below 0.1%. Prev, prevalence; CS, cross-sectional; Conv, convenience; RDS, respondent-driven sampling; SRS, simple random sampling.

The variance of the prevalence measures was stabilized using the Freeman–Tukey type arcsine square-root transformation [138]. Estimates for HCV prevalence were weighted by the inverse variance and pooled using a DerSimonian–Laird random-effects model. This model accounts for sampling variation (random chance) and expected heterogeneity in effect size across studies [139]. Heterogeneity was assessed and characterized using several statistical measures.

With a recently identified potential issue with the Freeman–Tukey type arcsine square-root transformation [140], we conducted sensitivity analyses by performing meta-analyses using the generalized linear mixed models (GLMM) method to confirm validity of our results.

Meta-analysis of RNA HCV prevalence measures among HCV antibody-positive individuals (that is HCV viraemic rate) was also conducted to estimate the pooled mean of this prevalence measure.

A sensitivity analysis was further performed to examine whether the advent of more specific and sensitive diagnostic tools (third or fourth generation assays) could have affected the prevalence estimates in the general population. Meta-analyses were performed on the general population prior to and after 2005, since after this year the vast majority of studies were likely to have been conducted using third of fourth generation assays. The results of the meta-analyses were assessed to determine whether the estimated pooled mean HCV prevalence was significantly different prior to 2005.

Meta-analyses were conducted in R v. 3.1.2. [141], using the package *meta* [142].

### 2.6. Quality assessment

The quality of HCV prevalence measures was assessed for each study as informed by the risk of bias (ROB) Cochrane approach [143], as well as by examining the precision of each reported measure. The ROB assessment was based on three domains: type of HCV ascertainment (biological assays versus unclear), the sampling methodology (probability-based versus convenience sampling) and the response rate (greater than or equal to 80% versus less than or equal to 80% of the target sample size).

Studies were considered as having high precision if the number of HCV tested individuals was at least 100 participants, as informed by previous studies [11–17].

## 3. Results

### 3.1. Search results

Figure 1 describes the process of study selection, adapted from the PRISMA flow diagram [26]. A total of 1375 citations were identified: 480 through PubMed and 895 through Embase. A total of 517 reports were identified as relevant or potentially relevant after removing duplicates and screening the titles and abstracts. Out of these, 285 reports were excluded for various reasons as summarized in figure 1. An additional report was identified through screening of articles' references, and 11 HCV prevalence measures/strata were obtained from the Pakistan National Survey [10]. Finally, 233 eligible reports were included in this systematic review, yielding one incidence study and 248 prevalence measures. The 248 prevalence measures contributed 341 prevalence measures/strata. Though no language restrictions were imposed, all identified studies were in English.

### 3.2. HCV incidence overview

Our search identified one HCV incidence study, which reported seroconversion risk. This study included (as its baseline) HCV-negative HCWs who reported a needle stick injury from documented HCV-positive patients. After six weeks follow-up, investigators reported a seroconversion risk of 4.8% [144].

### 3.3. HCV prevalence overview

#### 3.3.1. General population

Among the general population (table 1), our search identified 148 prevalence measures/strata, ranging from 0.4 to 44.0%, with a median of 5.3%. Among blood donors (number of studies; *n* = 57), HCV prevalence ranged from 0.4 to 20.8%, with a median of 3.5%. Among pregnant women (*n* = 12), HCV prevalence ranged from 0.7 to 20.7%, with a median of 6.0%. Among outpatients (*n* = 9), HCV prevalence ranged from 4.4 to 51.0%, with a median of 9.0%. Among other general populations (*n* = 65), HCV prevalence ranged from 0.4 to 35.9%, with a median of 6.8%.

#### 3.3.2. High-risk clinical populations

Among high-risk clinical populations (table 2), our search identified 21 prevalence measures/strata, ranging from 7.8 to 68.0%, with a median of 34.5%. Among thalassaemia patients (*n* = 12), HCV prevalence ranged from 7.7 to 60.0%, with a median of 42.2%. Among haemodialysis patients (*n* = 7), HCV prevalence ranged from 16.4 to 68.0%, with a median of 28.0%. Only one study was conducted for each of haemophilia patients (prevalence of 51.4%) and multi-transfused patients (prevalence of 54.2%).

#### 3.3.3. Intermediate risk populations

Among intermediate risk populations (electronic supplementary material, table S2), our search identified 64 prevalence measures/strata, ranging from 0.0 to 70.9%, with a median of 12.9%. Among hospitalized populations (*n* = 25), HCV prevalence ranged from 2.5 to 71.0%, with a median of 13.2%. Among HCWs (*n* = 11), HCV prevalence ranged from 0.0 to 5.6%, with a median of 3.2%. Among prisoners and/or volunteer prisoner blood donors (*n* = 9), HCV prevalence ranged from 8.7 to 18.2%, with a median of 13.1%. Among diabetics (*n* = 6), HCV prevalence ranged from 5.1 to 43.0%, with a median of 15.5%. Among household contacts of HCV index patients (*n* = 4), HCV prevalence ranged from 4.4 to 38.0%, with a median of 18.3%. A study conducted in Karachi among men who use roadside barbers measured HCV prevalence at 38.0% [145].

#### 3.3.4. Special clinical populations

Among special clinical populations (electronic supplementary material, table S3), our search identified 18 prevalence measures/strata, ranging from 1.0 to 81.0%, with a median of 15.5%. Among patients with skin disorders (*n* = 4), HCV prevalence ranged from 3.0 to 23.4%, with a median of 7.7%. Among patients with urological conditions (*n* = 4), HCV prevalence ranged from 1.0 to 25.9%, with a median of 9.6%.

#### 3.3.5. Populations with liver-related conditions

Among populations with liver-related conditions (electronic supplementary material, table S4), our search identified 73 prevalence measures/strata, ranging from 3.0 to 100.0%, with a median of 63.5%. Among chronic liver disease patients (*n* = 20), HCV prevalence ranged from 4.9 to 78.4%, with a median of 41.1%. Among cirrhosis patients (*n* = 21), HCV prevalence ranged from 28.0 to 100.0%, with a median of 68.0%. Among hepatocellular carcinoma patients (*n* = 18), HCV prevalence ranged from 33.3 to 92.0%, with a median of 70.1%. Among acute viral hepatitis patients (*n* = 6), HCV prevalence ranged from 6.4 to 57.1%, with a median of 20.9%.

#### 3.3.6. People who inject drugs

Among PWID (table 3), our search identified 15 prevalence measures/strata, ranging from 8.0 to 94.3%, with a median of 44.7%.

### 3.4. Overview of HCV RNA prevalence among HCV antibody-positive individuals

Our search identified a total of 12 HCV RNA prevalence measures among HCV antibody-positive individuals (HCV viraemic rate). The details of these measures can be found in the electronic supplementary material, table S6. HCV viraemic rate ranged from 44.4 to 98.0%, with a median of 74.2%.

### 3.5. Pooled mean HCV prevalence estimates

Pooled mean estimates for HCV prevalence for the six risk populations are summarized in table 4. The pooled mean prevalence for the general population (populations at low risk) was estimated at 6.2% (95% CI: 5.7–6.7%). Meanwhile, the pooled mean HCV prevalence was estimated at 34.5% (95% CI: 27.0–42.3%) for high-risk clinical populations, 12.8% (95% CI: 10.8–15.1%) for intermediate risk populations, 16.9% (95% CI: 6.2–31.3%) for special clinical populations, 55.9% (95% CI: 49.2–62.5%) for populations with liver-related conditions and 53.6% (95% CI: 36.2–70.6) for PWID.

**Table 4.** Pooled mean estimates for hepatitis C virus (HCV) prevalence for each of the six risk population categories in Pakistan.

	studies	samples	HCV prevalence		pooled HCV prevalence		heterogeneity measures		
risk population	total *n*	total *N*	range (%)	median (%)	mean (%)	95% CI	*Q* (*p*-value)^a^	*I*^2^ (confidence limits)^b^	prediction interval (%)^c^
general population (populations at low risk)	148	1 352 080	0.4–50.6	5.3	6.2	5.7–6.7	17 552.0 (<0.0001)	99.2% (99.1–99.2%)	1.7–13.0
high-risk clinical populations	21	2377	7.8–68.0	33.3	34.5	27.0–42.3	294.3 (<0.0001)	93.2% (90.9–94.9%)	5.5–72.0
populations at intermediate risk	64	156 623	0.0–70.9	12.9	12.8	10.8–15.1	8680.5 (<0.0001)	99.3% (99.2–99.3%)	1.2–33.7
special clinical populations	20	11 940	1.1–80.8	15.5	16.9	6.2–31.3	5666.9 (<0.0001)	99.7% (99.6–99.7%)	0.0–90.2
populations with liver-related conditions	73	23 132	3.1–100.0	63.5	55.9	49.2–62.5	7028.9 (<0.0001)	99.0% (98.9–99.1%)	6.7–98.2
PWID	15	2815	7.8–93.8	44.7	53. 6	36.2–70.6	1181.9 (<0.0001)	98.8% (98.6–99.0%)	0.0–100

^a^*Q*: the Cochran's *Q*-statistic, a measure assessing the existence of heterogeneity in effect size.

^b^*I*²: a measure assessing the magnitude of between-study variation that is due to differences in effect size across studies rather than chance.

^c^Prediction interval: estimates the 95% interval in which the true effect size in a new HCV study will lie.

Of note, the GLMM meta-analyses produced similar pooled mean estimates for all risk populations. For example, the pooled mean HCV prevalence for special clinical populations, that showed the largest difference between the fixed effects result and the random-effects result, was 13.1% (95% CI: 6.9–31.3) using the GLMM method versus 16.9% (95% CI: 6.2–31.3%) using the Freeman–Tukey type arcsine square-root transformation method.

Statistically significant heterogeneity in effect size (that is HCV prevalence) was observed in all meta-analyses (Cochrane's *Q*-statistic's *p*-value was always less than 0.0001; table 4). Most of the variation across pooled studies was due to true difference in effect size rather than chance (*I*^2^ > 93.7%). The prediction intervals were generally very wide. The totality of these heterogeneity measures indicates high heterogeneity in HCV prevalence measures in each risk population category.

The pooled mean HCV RNA prevalence among HCV antibody-positive individuals (HCV viraemic rate) was estimated at 74.1% (95% CI: 59.5–86.5%).

The meta-analyses performed prior to and after 2005 among the general population, as part of our sensitivity analysis, estimated a pooled mean HCV prevalence of 5.0% (95% CI: 4.0–6.0%), and 6.5% (95% CI: 5.9–7.0%), respectively.

### 3.6. Risk factors for HCV infection

Risk factors for HCV seropositivity were assessed in 11 studies using multivariable regression analyses. Healthcare-related risk factors were most commonly reported, including history of blood transfusions [54,71,146], dental work [51,71,147], surgery [54,71,146], medical injections [42,51,147] and being a HCW [87].

Injecting drug-use-related risk factors were also commonly reported, including history of injecting drug use [54,87,95,146,148], duration of injecting drug use [131], sharing of needles or syringes [54], source of needles or syringes [135] and ‘jerking’ (drawing blood into a syringe while injecting) [131]. Sexual risk factors were also reported, including sex work (females and males), and sex for drugs [146].

### 3.7. Quality assessment of HCV incidence and prevalence measures

Findings of the quality assessment are summarized in the electronic supplementary material, table S5. Only one study was identified for HCV incidence [144] (not shown in the electronic supplementary material, table S5), in which there were greater than or equal to 100 participants, and was therefore classified as having high precision. As it was based on convenience sampling, it had high ROB for this domain. Meanwhile, it had low ROB in HCV ascertainment and in the response rate domains.

The majority of HCV prevalence studies (86.7%) was based on samples with greater than or equal to 100 participants, and were therefore classified as having high precision. Most studies (67.7%) reported specific details about HCV ascertainment, but nearly 70% did not report the assay generation. When information was provided, 94.2% of studies reported use of third or fourth generation assays.

A sensitivity analysis was performed to assess whether HCV prevalence in the general population differed prior to and after 2005, because the vast majority of studies after this year were likely to have been conducted using third or fourth generation assays. The confidence intervals of the estimated pooled mean HCV prevalence prior to and after 2010 overlapped, indicating HCV prevalence was not significantly different between these two time durations.

The majority of HCV prevalence studies (92.3%) used convenience, non-probability-based sampling approach. Nearly half of studies had low ROB in the response rate domain and 48.8% had missing information—only 1.6% of studies had high ROB in this domain.

To summarize, 78.6% of studies had low ROB based on at least one domain, and 41.1% had low ROB based on at least two domains. Furthermore, 1.2% of studies had high ROB based on two domains, and no study had high ROB based on three domains. The totality of the quality assessment measures indicates reasonable study quality.

## 4. Discussion

We presented a systematic review and synthesis of HCV incidence and prevalence in Pakistan. Our results affirm that Pakistan has one of the highest HCV infection levels in both MENA [11–17] and worldwide [149–151]. HCV prevalence in the population at large is at about 5%—one in every 20 Pakistanis has been already exposed to HCV infection. HCV prevalence was also found to be high in all risk populations, testifying to the scale of the epidemic in this country. Our results further supported a major role for HCV infection in liver disease burden in Pakistan—over half of the populations with liver-related conditions were found HCV antibody-positive.

Our results collectively indicate a major role for healthcare in HCV transmission. High HCV prevalence was observed in the populations exposed to healthcare in one form or another. In high-risk clinical populations, the pooled mean HCV prevalence was high at 34.5% (95% CI: 27.0–42.3%) (table 4), with HCV prevalence ranging across studies from 7.8 to 68.0% (table 2)—much higher than that found in the general population. In special clinical populations, the pooled mean HCV prevalence was also high at 16.9% (95% CI: 6.2–31.3%) (table 4), with HCV prevalence ranging across studies from 1.0 to 81.0% (electronic supplementary material, table S3). In all identified reports on hospitalized populations, HCV prevalence ranged from 2.5 to 71.0%, with a median of 13.2% (electronic supplementary material, table S2).

Our assessment of HCV risk factors further indicates that HCV transmission appears to be primarily driven by healthcare-related exposures, such as therapeutic injections, intravenous infusions and poor sterilization of medical equipment [42,51,54,71,87]. Injecting drug use and other community-based exposures appear also to play a role, but their *relative* (as opposed to absolute) role is probably small compared with healthcare procedures [152]. These findings demonstrate the urgency of addressing the HCV epidemic in Pakistan, one of the world's largest, and where 10% of the global number of chronically infected people are living [6,21].

The apparent major role for healthcare in HCV transmission distinguishes Pakistan from most other countries. Though healthcare plays a role in both developing and developed countries [11–17,153–155], healthcare practices appear to have driven HCV prevalence to atypically high levels in this country, a pattern seen only in a limited number of countries globally, such as Egypt [13,22,23] and former Soviet republics [156]. This role for healthcare is not only manifested in the high HCV prevalence in the different clinical populations (table 4) and in the reported risk factors in analytical epidemiologic studies [42,51,54,71,87], but also in the outcomes of viral hepatitis surveillance [157]. For example, the recently established viral hepatitis surveillance system in Pakistan indicated that healthcare-related exposures appear to be behind most newly reported HCV viral hepatitis cases [157]. Importantly, the surveillance demonstrated also that HCV accounted for over half of reported viral hepatitis cases [157], highlighting the special role of HCV infection in viral hepatitis disease burden in this country.

Of healthcare exposures, unnecessary therapeutic injections and reuse of syringes and needles were highlighted often as key factors [157,158]. Pakistan has one of the highest rates of therapeutic injections worldwide [159,160]—with widespread perception that injectable medications are more effective than oral medications [161–163]. Financial incentives appear also to sustain this preference for injectable medications, as healthcare providers can charge more for medications when they are administered by injections [163]. Though Pakistan has attempted to enhance provision and use of disposable injections and passed regulations for the management of disposable medical devices [164], implementation has been challenging in a country where the private sector accounts for 70% of healthcare services [157,162,165]. It bears notice that despite a possible key role for therapeutic injections, the totality of the evidence synthesized in the present study suggests that HCV healthcare exposures occur through multiple and diverse healthcare procedures.

The regional context of Pakistan and drug trafficking routes [166] support a conducive environment for injecting drug use. Our results indicated a high HCV prevalence among PWID (table 4), and evidence for injecting drug use as a mode of HCV exposure [54,87,131,157]. However, with an estimate of only 104 804 active PWID in Pakistan [167–169], the *relative* contribution of injecting drug use to HCV incidence is probably substantially smaller than that of healthcare, although the exact quantitative contribution remains uncertain.

Our results highlight the urgent and immediate need for expansion of HCV treatment and prevention programmes in Pakistan. High HCV prevalence was observed among all risk populations (table 4), with about one in every 20 Pakistanis being infected. Furthermore, three-quarters of all HCV antibody-positive individuals in Pakistan, per the meta-analysis of HCV viraemic rate (Section: *Pooled mean HCV prevalence estimates*), are chronically infected with HCV and can transmit the infection further. In spite of heavily discounted prices for DAAs in Pakistan [170], treatment scale-up has been limited, with only 311 000 chronic infections treated since 2013 [171]. To reach the WHO global target of reducing incidence by 80% by 2030, a recent modelling study indicated that the annual number of treatments must reach 490 000 and be sustained at this level for at least a decade [24]. To address the alarmingly high burden of HCV and achieve WHO global targets by 2030, Pakistan has recently developed the first National Hepatitis Strategic Framework, emphasizing the scale-up of interventions in healthcare settings and of HCV screening and treatment as well as harm reduction services [172].

Our study has identified key gaps and weaknesses in HCV epidemiological evidence in Pakistan. Despite the large epidemic, only one (now outdated) nationally representative and probability-based population-based survey was conducted in this country [10]. Repeating and enhancing this survey is critical to assess trends in prevalence and risk factors, as well as potential changes in the epidemiology. Such surveys have played an instrumental role in elucidating our knowledge of HCV transmission and in informing HCV response in other countries, such as in Egypt [173–179] and the USA [180].

Despite the major role for healthcare, a relatively small number of studies have been conducted among clinical populations, or investigated healthcare-related exposures. This is to be contrasted, for example, with Iran where a large number of studies investigated the role of healthcare—despite the relatively small role of this mode of exposure in this country [16]. Hardly any analytical cohort studies have been conducted in Pakistan despite the large epidemic, in contrast to Egypt [13,22], another MENA country with a large HCV epidemic [23]. Despite some suggestive evidence for community-based exposures [152], such as visiting roadside barbers [161], this mode of exposures remains to be clarified with concrete analytical studies. Though HCV vertical transmission appears to account for a quarter of HCV infections among children under 5 years of age in Pakistan [181], only one study appears to have investigated this mode of exposure in this country [32].

Our study is limited by the quantity and quality of reviewed studies, as well as their representativeness of the different risk populations—most studies used convenience sampling as opposed to probability-based population-based sampling. Only PubMed and Embase databases were searched, but other HCV data may exist in unpublished (grey literature) form, or are published in non-indexed journals. There was extensive heterogeneity in HCV prevalence measures in each risk population—possibly because of variability within the specific studied subpopulation, geographical location, sex and age-group representation in the sample, sampling technique and participant recruitment, year of study and study quality.

Despite these limitations, the main strength of our study is that we identified a large number of studies that covered different risk populations, and that facilitated a comprehensive synthesis of evidence and identification of gaps and weaknesses that preclude a satisfactory understanding of HCV epidemiology in Pakistan.

## 5. Conclusion

Pakistan is enduring an HCV epidemic of historical proportions—one in every 20 Pakistanis has been already infected with this infection playing a major role in liver disease burden in this country. HCV prevalence is high in all risk populations with most transmission apparently driven by healthcare procedures. Though our knowledge of the specific modes of exposure that drive transmission is improving, our understanding is still hampered by key gaps and weaknesses in available evidence. Conduct of repeated and comprehensive nationally representative and probability-based population-based surveys is critical to assess HCV prevalence and trends, identify risk factors and modes of exposure, examine the spatial variability in prevalence, and assess HCV knowledge and attitudes.

HCV treatment and prevention must become a national priority in Pakistan. Although Pakistan has made efforts to increase coverage of safe injection and blood screening and to improve infection control [164,182–184], commitment to prevention in all segments of the healthcare system, including the private sector, should be secured for this country to accomplish the HCV elimination target by 2030. Major expansion of infection control in healthcare facilities, and of harm reduction services for PWID, are warranted, as well as adoption of the WHO guidelines for the use of safety-engineered syringes [185,186].

## Supplementary Material

Supplementary Material

## Data accessibility

The datasets supporting this article have been uploaded as part of the manuscript and electronic supplementary material.
