# Supplementary material for: The epidemiology of hepatitis C virus in Pakistan: systematic review and meta-analyses
Source: R Soc Open Sci. 2018 Apr 11;5(4):180257. doi: 10.1098/rsos.180257 (PMC5936963; doi:10.1098/rsos.180257)
Supplement: Supplementary Material [file rsos180257supp1.docx]

**SUPPLEMENTARY MATERIAL**

**The epidemiology of hepatitis C virus in Pakistan: Systematic review and meta-analyses**

Zaina Al Kanaani,^1^ Sarwat Mahmud,^1^ Silva P. Kouyoumjian,^1^ and Laith J. Abu-Raddad^1, 2,*^

^1^*Infectious Disease Epidemiology Group, Weill Cornell Medicine-Qatar, Qatar Foundation - Education City, Doha, Qatar*

^2^*Department of Healthcare Policy & Research, Weill Cornell Medicine, Cornell University, New York, USA*

***Reprints or correspondence:** Prof. Laith J. Abu-Raddad, Infectious Disease Epidemiology Group, Weill Cornell Medicine-Qatar, Qatar Foundation - Education City, P.O. Box 24144, Doha, Qatar. Telephone: + (974) 4492-8321. Fax: + (974) 4492-8333. E-mail: lja2002@qatar-med.cornell.edu

**Table S1.** Preferred Reporting Items for Systematic Reviews and Meta-analyses (PRISMA) checklist [1].

| **Section/topic** | **#** | **Checklist item** | **Reported in main text on** |
| --- | --- | --- | --- |
| **TITLE** | | |  |
| Title | 1 | Identify the report as a systematic review, meta-analysis, or both. | p. 1 |
| **ABSTRACT** | | |  |
| Structured summary | 2 | Provide a structured summary including, as applicable: background; objectives; data sources; study eligibility criteria, participants, and interventions; study appraisal and synthesis methods; results; limitations; conclusions and implications of key findings; systematic review registration number. | p. 2 |
| **INTRODUCTION** | | |  |
| Rationale | 3 | Describe the rationale for the review in the context of what is already known. | p. 4 |
| Objectives | 4 | Provide an explicit statement of questions being addressed with reference to participants, interventions, comparisons, outcomes, and study design (PICOS). | p. 4 |
| **METHODS** | | |  |
| Protocol and registration | 5 | Indicate if a review protocol exists, if and where it can be accessed (e.g., Web address), and, if available, provide registration information including registration number. | p. 4 |
| Eligibility criteria | 6 | Specify study characteristics (e.g., PICOS, length of follow-up) and report characteristics (e.g., years considered, language, publication status) used as criteria for eligibility, giving rationale. | p. 4-5 |
| Information sources | 7 | Describe all information sources (e.g., databases with dates of coverage, contact with study authors to identify additional studies) in the search and date last searched. | p. 4 and Figure S1 in SM |
| Search | 8 | Present full electronic search strategy for at least one database, including any limits used, such that it could be repeated. | Figure S1 in SM |
| Study selection | 9 | State the process for selecting studies (i.e., screening, eligibility, included in systematic review, and, if applicable, included in the meta-analysis). | p. 4-5 and Figure 1 |
| Data collection process | 10 | Describe method of data extraction from reports (e.g., piloted forms, independently, in duplicate) and any processes for obtaining and confirming data from investigators. | p. 5-7 |
| Data items | 11 | List and define all variables for which data were sought (e.g., PICOS, funding sources) and any assumptions and simplifications made. | p. 5-7 |
| Risk of bias in individual studies | 12 | Describe methods used for assessing risk of bias of individual studies (including specification of whether this was done at the study or outcome level), and how this information is to be used in any data synthesis. | p. 8 |
| Summary measures | 13 | State the principal summary measures (e.g., risk ratio, difference in means). | p. 5 |
| Synthesis of results | 14 | Describe the methods of handling data and combining results of studies, if done, including measures of consistency (e.g., I^2^) for each meta-analysis. | p. 7-8 |
| Risk of bias across studies | 15 | Specify any assessment of risk of bias that may affect the cumulative evidence (e.g., publication bias, selective reporting within studies). | p. 8 |
| Additional analyses | 16 | Describe methods of additional analyses (e.g., sensitivity or subgroup analyses, meta-regression), if done, indicating which were pre-specified. | p. 8 |
| **RESULTS** | | |  |
| Study selection | 17 | Give numbers of studies screened, assessed for eligibility, and included in the review, with reasons for exclusions at each stage, ideally with a flow diagram. | p. 8-9 and Figure 1 |
| Study characteristics | 18 | For each study, present characteristics for which data were extracted (e.g., study size, PICOS, follow-up period) and provide the citations. | p. 9-11 and Tables1-4 and Tables S2-S4 in SM |
| Risk of bias within studies | 19 | Present data on risk of bias of each study and, if available, any outcome level assessment (see item 12). | p. 13-14, and Table S5 in SM |
| Results of individual studies | 20 | For all outcomes considered (benefits or harms), present, for each study: (a) simple summary data for each intervention group (b) effect estimates and confidence intervals, ideally with a forest plot. | p. 13-14, Tables 1-4 and Figure S2-S7 in SM |
| Synthesis of results | 21 | Present results of each meta-analysis done, including confidence intervals and measures of consistency. | p. 11-12, Table 4 and Figure S2-S7 in SM |
| Risk of bias across studies | 22 | Present results of any assessment of risk of bias across studies (see Item 15). | p. 12, Table 4 |
| Additional analysis | 23 | Give results of additional analyses, if done (e.g., sensitivity or subgroup analyses, meta-regression [see Item 16]). | p. 12 |
| **DISCUSSION** | | |  |
| Summary of evidence | 24 | Summarize the main findings including the strength of evidence for each main outcome; consider their relevance to key groups (e.g., healthcare providers, users, and policy makers). | p. 14-18 |
| Limitations | 25 | Discuss limitations at study and outcome level (e.g., risk of bias), and at review-level (e.g., incomplete retrieval of identified research, reporting bias). | p. 18 |
| Conclusions | 26 | Provide a general interpretation of the results in the context of other evidence, and implications for future research. | p. 18-19 |
| **FUNDING** | | |  |
| Funding | 27 | Describe sources of funding for the systematic review and other support (e.g., supply of data); role of funders for the systematic review. | p. 20 |

**Figure S1.** Search criteria for systematically reviewing hepatitis C virus (HCV) incidence and prevalence data in Pakistan.

**PubMed (last searched: April 18, 2016)**

("Hepatitis C"[Mesh] OR "Hepatitis C Antibodies"[Mesh] OR "Hepatitis C Antigens"[Mesh] OR "Hepacivirus"[Mesh] OR "Hepatitis C"[Text] OR "Hepacivirus"[Text] OR "HCV"[Text]) AND ("Pakistan"[Mesh] or Pakistan*[text])

**Embase (last searched: April 19, 2016)**

(esp. Pakistan/ OR Pakistan*.mp.) AND (exp Hepatitis C/ OR hepatitis C.mp. OR exp hepatitis C antibody/ OR exp hepatitis C antigen/ OR exp Hepatitis C virus/ OR HCV.mp. OR hepacivirus .mp.)

**Table S2.** Studies reporting hepatitis C virus (HCV) prevalence among populations at intermediate risk in Pakistan.

| Author, year (citation) | Year(s)  of data  collection | Province or city | Study site | Study  design | Study  Sampling  procedure | Population | Sample size* | HCV  prev** |
| --- | --- | --- | --- | --- | --- | --- | --- | --- |
| Mujeeb, 1998 [2] | - | Karachi | - | CS | Conv | Healthcare workers | 114 | 4.4% |
| Pasha, 1999 [3] | 1993-94 | Hafizabad | Community | CS | Conv | Household contacts of index patients | 74 | 16.0% |
| Aziz, 2002 [4] | - | Karachi | Hospital | CS | Conv | Healthcare workers | 250 | 5.6% |
| Qureshi, 2002 [5] | 1996-99 | Karachi | Diabetes clinic | CS | Conv | Diabetic patients | 196 | 5.1% |
| Akhtar, 2004 [6] | 1999 | Karachi | Community | CS | Conv | Household contacts of index patients | 341 | 20.5% |
| Ally, 2005 [7] | 2004 | Abbottabad | Hospital | CS | Conv | Outpatients and inpatients | 4,638 | 5.8% |
| Khokhar, 2005 [8] | 2001-04 | Islamabad | Hospital | CS | Conv | Spouses of index patients | 227 | 4.4% |
| Abbas, 2007 [9] | 2006 | Rural area | Medical center | CS | Conv | Inpatients | 55 | 71.0% |
| Ali, 2007 [10] | - | Peshawar | Hospital | CS | Conv | Diabetic patients | 100 | 36.0% |
| Khan, 2007 [11] | 2003-04 | Abbottabad | Hospital | CS | Conv | Inpatients | 1,630 | 3.3% |
| Qureshi, 2007 [12] | - | - | Medical center | CS | Conv | Spouses of index patients | 153 | 38.0% |
| Tariq, 2007 [13] | 2007 | Faisalabad | Hospital | CS | Conv | Inpatients (ophthalmic procedures) | 1,027 | 27.1% |
| Daudpota, 2008 [14] | 2007 | Jacobabad Sindh | Medical center | CS | Conv | Inpatients: >13 years old | 150 | 14.0% |
| Sarwar, 2008 [15] | 2006-07 | Abbottabad | Hospital | CS | Conv | Healthcare workers | 125 | 5.6% |
| Sami, 2009 [16] | 2005 | Karachi | Medical center | CS | Conv | Inpatients (gynecological procedures) | 584 | 16.0% |
| Zuberi, 2009 [17] | 2004-08 | Karachi | University | Pros C | Conv | Healthcare workers | 215 | 2.3% |
| Gorar, 2010 [18] | 2008-09 | Sindh | Prisons | CS | Conv | Prisoners | 7,539 | 12.8% |
| Hussain, 2010 [19] | 2009 | - | Diabetes clinic | CS | Conv | Diabetic patients | 100 | 43.0% |
| Jadoon, 2010 [20] | 2008 | Multan | Hospital | CS | Conv | Diabetic patients | 550 | 15.6% |
| Jadoon, 2010 [21] | - | Multan | Hospital | CC | Conv | Diabetic patients | 3,000 | 13.7% |
| Kazi, 2010 [22] | 2007-08 | Karachi | Prison | CS | SsCS | Male prisoners | 357 | 18.2% |
| Majid, 2010 [23] | 2004-08 | Bannu, Khyber-Pakhtunkhwa | Hospital | CS | Conv | Outpatients and inpatients : >15 years old | 25,944 | 3.3% |
| Makheja, 2010 [24] | 2007-08 | Karachi | Medical center | CS | Conv | Men who visit roadside barbers | 184 | 38.0% |
| Memon, 2010 [25] | 2009-10 | Sukkur | Hospital | CS | Conv | Inpatients | 913 | 13.8% |
| Khan, 2011 [26] | - | Peshawar, Khyber-Pakhtunkhwa | Hospitals | CS | Conv | Health care workers | 824 | 4.1% |
| Naeem, 2011 [27] | 2010-11 | Karachi | Hospital | CS | Conv | Inpatients (cataract operation) | 377 | 11.1% |
| Iqbal, 2012 [28] | 2011 | Lahore | Hospital | CS | Conv | Inpatients (admitted for elective surgery) | 1,185 | 14.0% |
| Memon, 2012 [29] | 2007-08 | Karachi | Hospital | CS | Conv | Health care workers | 1,051 | 2.9% |
| Memon, 2012[29] | 2007-08 | Karachi | Prison | CS | Conv | Prisoners | 2,287 | 8.7% |
| Muhammad, 2012 [30] | 2009-10 | Sindh | Medical center | CC | Conv | Inpatients | 584 | 8.6% |
| Pervaiz, 2012 [31] | 2009-10 | Punjab | Prisons | CS | Conv | Male prisoner blood donors | 41,923 | 13.1% |
| Pervaiz, 2012 [31] | 2009-10 | Punjab | Prisons | CS | Conv | Female prisoner blood donors | 1,120 | 16.0% |
| Qayyum, 2012 [32] | 2011 | Lahore | Hospital | CS | Conv | Inpatients and outpatients | 1,891 | 16.6% |
| Bota, 2013 [33] | - | Karachi | Hospital | CS | Conv | Inpatients | 662 | 6.8% |
| Latif, 2013 [34] | 2013 | Faisalabad | Hospital | CS | Conv | Inpatients (cataract surgery) | 379 | 47.6% |
| Qadeer, 2013 [35] | 2007-12 | Punjab | Prisons | CS | Conv | Prisoner blood donors | 5,000 | 12.3% |
| Maan, 2014 [36] | 2010-12 | Faisalabad | Hospital | CS | Conv | Patients a attending STD clinic | 39,780 | 22.0% |
| Shams, 2014 [37] | 2012-13 | Karachi | Hospital | CS | Conv | Patients planned for elective surgery | 107 | 10.2% |
| Asad, 2015 [38] | 2013 | Farah town, Islamabad | Laboratory in a hospital | CS | Conv | Inpatients and outpatients | 345 | 33.0% |
| Fayyaz, 2015 [39] | 2014 | Abbottabad | Dental clinic in a hospital | CS | Conv | Patients attending dental unit | 3,549 | 2.8% |
| Pervaiz, 2015 [40] | 2007-09 | Punjab | Prisons | CS | Conv | Prisoner blood donors | 5,894 | 15.2% |
| Saqib, 2016 [41] | 2014 | Islamabad & Rawalpindi | Hospitals | CS | SRS | Healthcare workers | 500 | 1.8% |

*The table reports only studies whose sample size is ≥50 participants. For space considerations, the table shows the overall HCV measure of each study rather than stratifications within population subgroups.
**The decimal places of the prevalence figures are as reported in the original report, but prevalence figures with more than one decimal were rounded to one decimal place, with the exception of those below 0.1%. Abbreviations: Prev, prevalence; CS, cross-sectional; CC, case-control; Pros C, prospective cohort; Conv, convenience; SsCS, single-stage cluster sampling; SRS, simple random sampling; STD, sexually transmitted. diseases.

**Table S3.** Studies reporting hepatitis C virus (HCV) prevalence among special clinical and mixed populations in Pakistan.

| Author, year (citation) | Year(s) of  data  collection | Province or city | Study site | Study design | Study  Sampling  procedure | Population | Sample  size* | HCV prev** |
| --- | --- | --- | --- | --- | --- | --- | --- | --- |
| Special clinical populations (number of studies=18) | | | | | |  |  |  |
| Adil, 2001 [42] | 1990-96 | Karachi | - | CS | Conv | Aplastic anemia patients | 144 | 2.8% |
| Mahboob, 2003 [43] | 1999-01 | Lahore | Hospital | CS | Conv | Lichen planus patients | 184 | 23.4% |
| Nasreen, 2007 [44] | 2005 | Karachi | Hospital | CS | Conv | Lichen planus patients | 63 | 6.3% |
| Naqvi, 2008 [45] | 2007 | Punjab | Community | CS | Conv | Kidney vendors (post nephrectomy) | 104 | 25.9% |
| Naqvi, 2008 [45] | 1996-07 | Punjab | Dialysis center | CS | Conv | Kidney donors (post nephrectomy) | 184 | 1.0% |
| Batool, 2009 [46] | 2007-08 | Lahore | Laboratory | CS | Conv | Patients with unidentified illness | 2,000 | 6.5% |
| Waheed, 2010 [47] | 2005-09 | Lahore | Hospital | CS | Conv | Patients who underwent different urological procedures | 558 | 13.4% |
| Qazi, 2011 [48] | 2005-06 | Bahawalpur | Hospital | CS | Conv | Patients with dyspepsia | 2,200 | 16.8% |
| Alvi, 2012 [49] | 2010-11 | Malir district | Hospital | CS | Conv | Patients who reported to the hospital in a year | 2,093 | 20.4% |
| Habib, 2012 [50] | - | Pano Aqil | Hospital | CS | Conv | Patients with vitiligo | 230 | 3.0% |
| Muhammad, 2012 [30] | 2009-10 | Sindh | Medical center | CC | Conv | Non-Hodgkin Lymphoma patients | 292 | 17.8% |
| Rahman, 2012 [51] | 2003-05 | Karachi | Hospital | CS | Conv | Patients with skin disease | 355 | 9.0% |
| Ahmad, 2013 [52] | 2012 | Karachi | Hospital | CS | Conv | Patients who underwent endoscopy( biopsies and resections) | 129 | 44.2% |
| Butt, 2014 [53] | 2013-14 | Karachi | Medical center | CS | Conv | Patients with esophagogastric varices | 99 | 81.0% |
| Imran, 2014 [54] | 2005-14 | Multan | - | CS | Conv | Children with nephrotic syndrome (biopsies & records) | 80 | 1.3% |
| Taj, 2014 [55] | 2003-08 | Karachi | Hospital | CS | Conv | Aplastic anemia Patients | 318 | 2.2% |
| Xu, 2014 [56] | 2011 | Southern Punjab | Hospital | CS | Conv | Euthyroid patients | 120 | 15.0% |
| Shakeel, 2015 [57] | 2010-14 | Lahore | Hospital | CS | Conv | Patients with esophageal varices | 3,392 | 79.0% |
| Mixed populations (number of studies=5) | | | |  |  |  |  |  |
| Khan, 2008 [58] | 2005-06 | Northern Pakistan | Community | CS | Conv | Patients and paramedical staff | 245 | 3.3% |
| Khan, 2008 [58] | 2006 | Northern Pakistan | Community | CS | Conv | Patients and paramedical staff | 290 | 5.9% |
| Abdullah, 2011 [59] | 2009-10 | Karachi | Diagnostic lab | CS | Conv | Children, pregnant women, blood donors, students, and health workers hospitalized patients, and those with disturbed or normal liver function enzymes. | 35,191 | 9.0% |
| Abdullah, 2011 [59] | 2002-07 | Karachi | Diagnostic lab | CS | Conv | Children, pregnant women, blood donors, students, and health workers | 37,391 | 5.9% |
| Ali, 2011 [60] | - | Khyber Pakhtunkhwa | - | CS | Conv | Thalassemia patients, dialysis patients, inpatients and injecting drug users | 167 | 15.6% |

*The table reports only studies whose sample size is ≥50 participants. For space considerations, the table shows the overall HCV measure of each study rather than stratifications within population subgroups.
**The decimal places of the prevalence figures are as reported in the original report, but prevalence figures with more than one decimal were rounded to one decimal place, with the exception of those below 0.1%.
Abbreviations: Prev, prevalence; CC, case-control; CS, cross-sectional; Conv, convenience.

**Table S4.** Studies reporting hepatitis C virus (HCV) prevalence among populations with liver-related conditions in Pakistan.

| Author, year (citation) | Year(s) of  data  collection | | Province or city | Study site | Study  design | Study  Sampling  procedure | | Population | Sample size* | HCV prev** |
| --- | --- | --- | --- | --- | --- | --- | --- | --- | --- | --- |
| Haider, 1994 [61] | | 1991 | Lahore | Hospital | CS | | Conv | Acute viral hepatitis patients | 93 | 6.4% |
| Ahmad, 1996 [62] | | 1987-92 | Rawalpindi | Hospital | CS | | Conv | Hepatocellular carcinoma & chronic hepatitis patients  hepatitis patients | 105 | 34.3% |
| Mujeeb, 1997 [63] | | 1989-92 | Karachi | Medical center | CS | | Conv | Hepatocellular carcinoma patients | 54 | 33.3% |
| Farooqi, 2000 [64] | | 1995-98 | Peshawar | Hospital | CS | | Conv | Cirrhosis patients | 410 | 50.0% |
| Farooqi, 2000 [65] | | 1995-98 | NWFP | Hospital | CS | | Conv | Hepatocellular carcinoma patients | 56 | 67.9% |
| Durrani, 2001 [66] | | 1997-00 | Balochistan | Hospital | CS | | Conv | Chronic liver disease patients | 160 | 40.0% |
| Farooqi, 2001 [67] | | 1996-00 | Peshawar | Medical center | CS | | Conv | Cirrhosis patients | 115 | 63.5% |
| Sharief, 2001 [68] | | 1994-98 | Southern Pakistan | - | CS | | Conv | Hepatocellular carcinoma patients | 133 | 57.0% |
| Aman, 2002 [69] | | 1997-00 | Lahore | Hospital | CS | | Conv | Hepatocellular carcinoma patients | 118 | 78.0% |
| Khan, 2002 [70] | | 1997 | Lahore | Hospital | CS | | Conv | Cirrhosis patients | 94 | 68.0% |
| Qureshi, 2002 [5] | | 1996-99 | Karachi | Medical research center | CS | | Conv | Chronic liver disease patients | 400 | 75.5% |
| Bukhtiari, 2003 [71] | | 1999-00 | Rawalpindi | Hospital | CS | | Conv | Chronic liver disease patients | 97 | 64.9% |
| Khan, 2003 [72] | | 2000-02 | Hazara Division | Private clinic | CS | | Conv | Chronic liver disease patients | 614 | 40.9% |
| Khokhar, 2003 [73] | | - | Islamabad | Hospital | CS | | Conv | Hepatocellular carcinoma patients | 67 | 67.0% |
| Nadeem, 2005 [74] | | 2002-03 | Karachi | Medical center | CS | | Conv | Cirrhosis patients | 170 | 28.0% |
| Maqsood, 2006 [75] | | 2005-05 | Islamabad | Medical center | CS | | Conv | Hepatic encephalopathy patients | 50 | 66.0% |
| Zuberi, 2006 [76] | | 2003-05 | Karachi | Hospital | CS | | Conv | Hepatitis B patients | 246 | 17.1% |
| Yusuf, 2007 [77] | | 1995-04 | Lahore | Registry | CS | | Conv | Hepatocellular carcinoma patients | 584 | 43.5% |
| Ali, 2008 [78] | | 2005-06 | - | Tertiary care center | CS | | Conv | Chronic liver disease patients | 176 | 60.2% |
| Almani, 2008 [79] | | 2005-07 | Hyderabad | Hospital | CS | | Conv | Cirrhosis patients | 100 | 68.0% |
| Rahopoto, 2008 [80] | | 2006-07 | Jamshoro | - | CS | | Conv | Cirrhosis patients | 200 | 76.5% |
| Abbas, 2009 [81] | | 2005-08 | Sindh | Liver clinic | CS | | Conv | Chronic liver disease patients | 1,638 | 20.0% |
| Ansari, 2009 [82] | | 2005-08 | Sindh | Hospital | CS | | Conv | Hepatocellular carcinoma patients | 200 | 77.5% |
| Bhatti, 2009 [83] | | - | Lahore | Hospital | CS | | Conv | Hepatocellular carcinoma patients | 276 | 76.5% |
| Devrajani, 2009 [84] | | 2007 | Jamshoro | Hospital | CS | | Conv | Hepatic encephalopathy patients | 87 | 65.0% |
| Idrees, 2009 [85] | | 2001-09 | Punjab & NWFP | Community | CS | | Conv | Hepatocellular carcinoma patients | 145 | 76.5% |
| Khan, 2009 [86] | | 2006 | Swat | Hospital | CS | | Conv | Cirrhosis patients | 110 | 63.6% |
| Soomro, 2009 [87] | | 2008-09 | Hyderabad | Hospital | CS | | Conv | Cirrhosis patients | 127 | 94.0% |
| Ahmed, 2010 [88] | | 2006-08 | Karachi | Hospital | CS | | Conv | Cirrhosis patients | 360 | 78.0% |
| Ahmed, 2010 [89] | | 1987-07 | Karachi | Medical center | CS | | Conv | Acute hepatitis patients | 346 | 6.7% |
| Ahmed, 2010 [89] | | 1987-07 | Karachi | Medical center | CS | | Conv | Cirrhosis patients | 1,430 | 59.4% |
| Kumar, 2010 [90] | | - | Tando Muhammad Khan city, Sindh, Pakistan | Laboratory | CS | | Conv | Suspected hepatitis patients | 5,989 | 17.0% |
| Mehmood, 2011 [91] | | 2006 | Peshawar | Hospital | CS | | Conv | Cirrhosis patients | 280 | 51.8% |
| Safdar, 2011 [92] | | 2010-11 | Islamabad | Hospitals | CS | | Conv | Viral hepatitis patients | 715 | 57.1% |
| Abbasi, 2011 [93] | | 2009-10 | Karachi | Medical center | CS | | Conv | Cirrhosis patients | 217 | 73.3% |
| Khan, 2011 [94] | | 2007-08 | Karachi | Outpatients and inpatients clinics | CS | | Conv | Acute liver disease patients | 65 | 3.0% |
| Mandokhel, 2011 [95] | | 2008-09 | Quetta | Hospital | CS | | Conv | Chronic liver disease patients | 1,054 | 21.5% |
| Nawaz, 2011 [96] | | 2008-11 | Punjab | Specialized clinic | CS | | Conv | Hepatocellular carcinoma patients | 84 | 92.0% |
| Tahir, 2011 [97] | | 2010-11 | Islamabad | Hospitals | CS | | Conv | Children with liver disease | 60 | 31.7% |
| Phulpoto, 2012 [98] | | 2008-10 | Northern Sindh | Hospitals | CS | | Conv | Cirrhosis patients (with no hepatocellular carcinoma) | 194 | 34.0% |
| Brohi, 2013 [99] | | 2009-10 | Hyderabad | Hospital | CS | | Conv | Pregnant women with acute liver failure | 52 | 13.5% |
| Butt, 2013 [100] | | 1999-09 | Karachi | Hospital | CS | | Conv | Hepatocellular carcinoma patients | 546 | 57.3% |
| Khan, 2013 [101] | | 2010-12 | Rawalpindi | Specialized clinic | CS | | Conv | Hepatocellular carcinoma patients | 199 | 88.4% |
| Memon, 2013 [102] | | 2005-10 | Hyderabad | Hospital | CS | | Conv | Hepatocellular carcinoma patients | 371 | 82.4% |
| Naqvi, 2013 [103] | | 2009-10 | Karachi | Hospital | CS | | Conv | Cirrhosis patients | 298 | 54.4% |
| Parkash, 2013 [104] | | - | Karachi | Hospital | CS | | Conv | Cirrhosis patients | 225 | 68.5% |
| Sundus, 2013 [105] | | 2009-10 | Karachi | Liver center in a hospital | CS | | Conv | Patients with hepatitis | 165 | 93.3% |
| Ubaid, 2013 [106] | | 2011-12 | Karachi | Liver transplant center | CS | | Conv | Chronic liver disease patients | 225 | 78.0% |
| Dar, 2014 [107] | | 2012-13 | - | Medical center | CS | | Conv | Liver donor liver transplant patients | 56 | 75.0% |
| Khan, 2014 [108] | | 2011-11 | Islamabad | - | CS | | Conv | Suspected viral hepatitis patients | 845 | 24.8% |
| Munaf, 2014 [109] | | 2009-13 | Hyderabad | Hospital | CS | | Conv | Hepatocellular carcinoma patients | 188 | 66.0% |
| Naz, 2014 [110] | | 2012-13 | Hyderabad | Hospital | CS | | Conv | Chronic liver disease patients | 150 | 78.4% |
| Qureshi, 2014 [111] | | 2011-13 | Islamabad | Hospital | CS | | Conv | End stage liver disease patients | 523 | 58.0% |
| Shah, 2014 [112] | | 2007-11 | Karachi | Hospitals | RCT | | Conv | Cirrhosis patients | 82 | 78.0% |
| Shah, 2014 [112] | | 2007-11 | Karachi | Hospitals | RCT | | Conv | Cirrhosis patients | 86 | 77.4% |
| Dar, 2015 [113] | | 2012-14 | - | - | CS | | Conv | Liver donor liver transplant patients | 100 | 73.0% |
| Haque, 2015 [114] | | 2012-14 | Lahore | Hospitals | CS | | Conv | Chronic liver disease patients | 253 | 58.1% |
| Khokhar, 2015 [115] | | 2012-14 | Islamabad | Hospital | RCT | | Conv | Chronic liver disease patients | 306 | 75.8% |
| Malik, 2015 [116] | | - | Rawalpindi | Hospital | CS | | Conv | Cirrhosis patients | 60 | 100.0% |
| Qureshi, 2015 [117] | | 2012-14 | Islamabad | Hospital | RCT | | Conv | Chronic liver disease patients | 306 | 75.8% |
| Sarwar, 2015 [118] | | 2007-13 | Lahore | Hospital | CS | | Conv | Hepatocellular carcinoma patients | 228 | 82.9% |
| Sunder, 2015 [119] | | - | - | Community | CS | | Conv | Viral hepatitis patients | 95 | 28.5% |

*The table reports only studies whose sample size is ≥50 participants. For space considerations, the table shows the overall HCV measure of each study rather than stratifications within population subgroups.
**The decimal places of the prevalence figures are as reported in the original report, but prevalence figures with more than one decimal were rounded to one decimal place, with the exception of those below 0.1%. Abbreviations: Prev; prevalence; NWFP, North West Frontier Province; CS, cross-sectional; RCT, randomized controlled trial; Conv, convenience.

**Table S5.** Summary of precision and risk of bias assessment for hepatitis C virus (HCV) prevalence measures extracted from eligible reports.

| **Quality assessment** | **HCV prevalence measures** | |
| --- | --- | --- |
|  | **Number of studies** | **%** |
| **Precision of estimates** |  |  |
| High precision | 215 | 86.7 |
| Low precision | 33 | 13.3 |
| **Risk of bias quality domains** |  |  |
| **Sampling methodology** |  |  |
| Low risk of bias | 19 | 7.7 |
| High risk of bias | 229 | 92.3 |
| **HCV ascertainmentǂ** |  |  |
| Low risk of bias | 168 | 67.7 |
| Unclear^*^ | 80 | 32.3 |
| **Response rate** |  |  |
| Low risk of bias | 124 | 49.2 |
| High risk of bias | 4 | 1.6 |
| Unclear^*^ | 120 | 48.8 |
| **Total number of studies where risk of bias assessment was possible** | **248** | **100** |
| **Total number of studies** | **248** | **100** |
| **Summary of risk of bias assessment for HCV prevalence Number of studies %**  **studies** | | |
| **Low risk of bias** |  |  |
| In at least one quality domain | 195 | 78.6 |
| In at least two quality domains | 102 | 41.1 |
| In all three quality domains | 13 | 5.2 |
| **High risk of bias** |  |  |
| In at least one quality domain | 230 | 92.7 |
| In at least two quality domains | 3 | 1.2 |
| In all three quality domains | 0 | 0 |
| **Total number of studies where risk of bias assessment was possible** | **248** | **100** |
| **Total number of studies** | **248** | **100** |

^*^Studies with missing information for any of the domains were classified as having unclear ROB for that specific domain.
ǂAll reports in which HCV was ascertained using self-report were excluded, therefore there is no high risk of bias category in this domain.

**Figure S2.** Forest plot of studies reporting hepatitis C virus (HCV) prevalence among the general population (populations at low risk) in Pakistan.


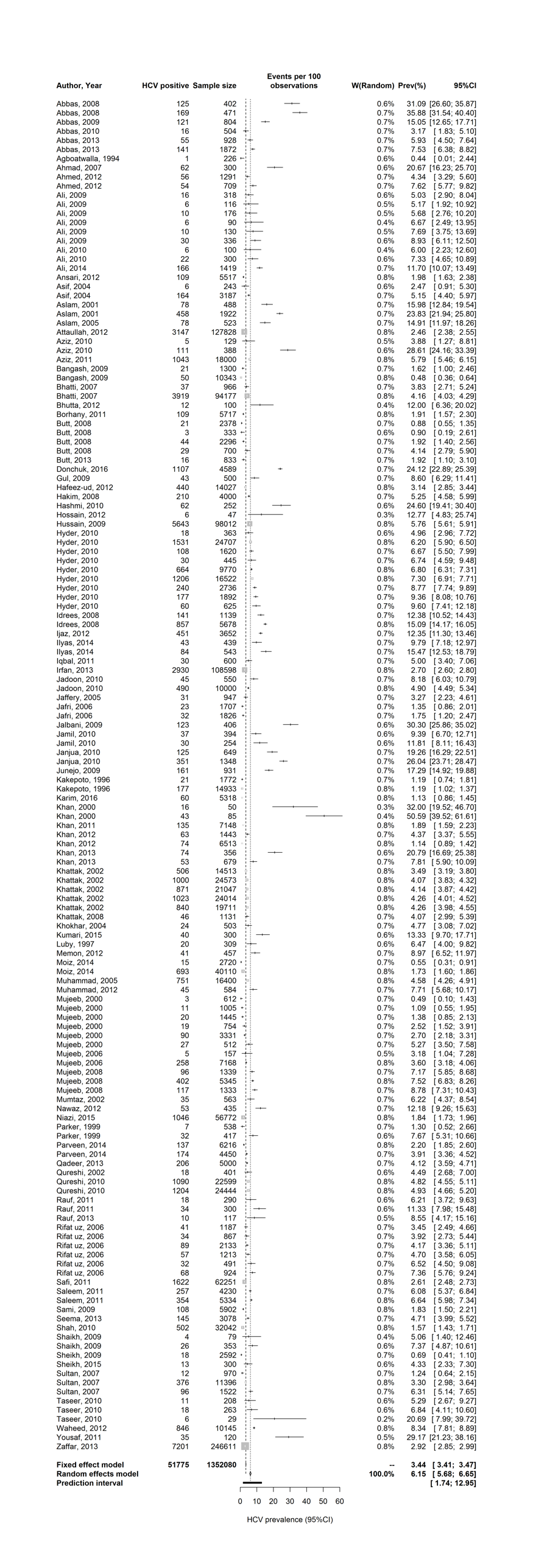


**Continuation of Figure S2a.**


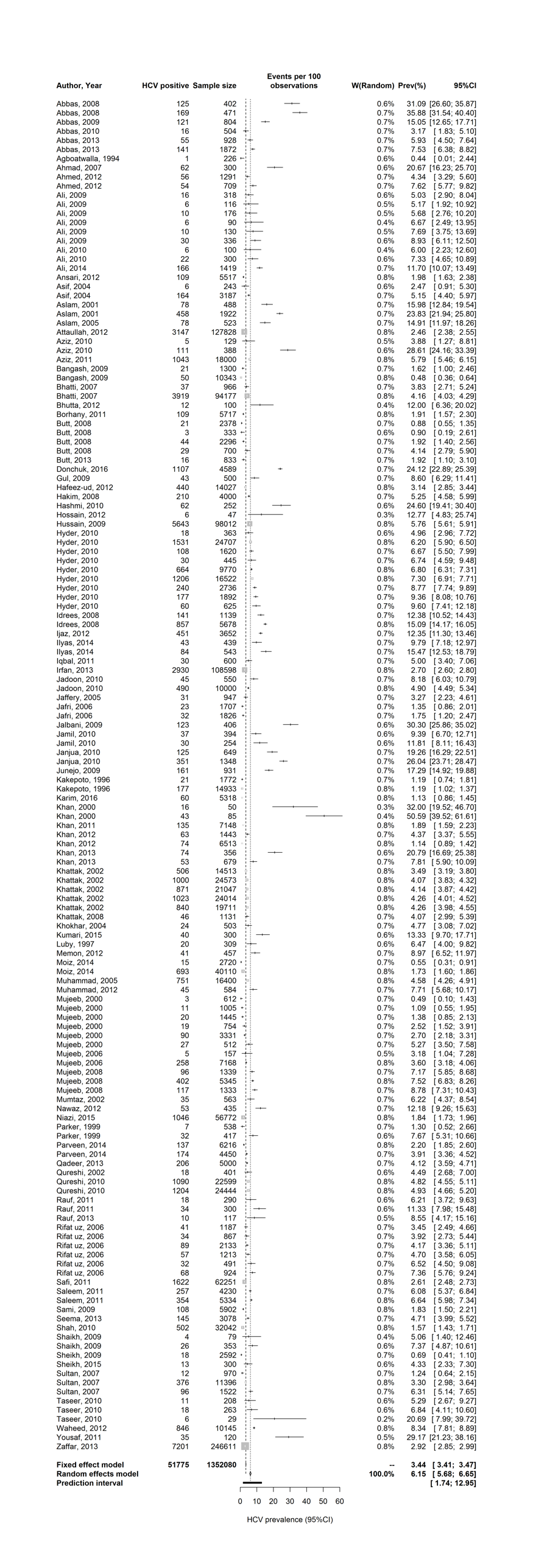


**
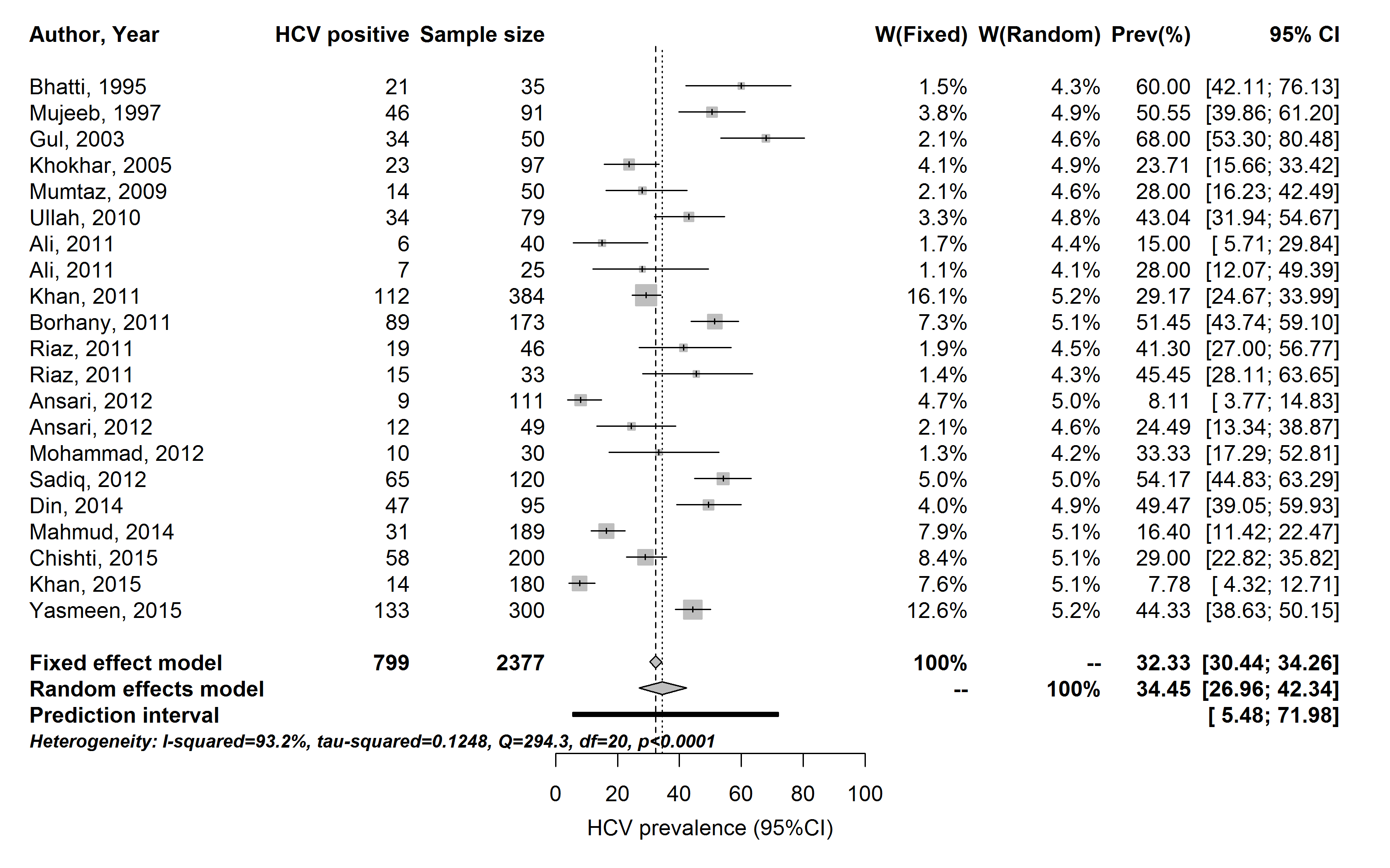
Figure S3.** Forest plot of studies reporting hepatitis C virus (HCV) prevalence among high risk clinical populations in Pakistan.

**Figure S4.** Forest plot of studies reporting hepatitis C virus (HCV) prevalence among intermediate risk populations in Pakistan.

**
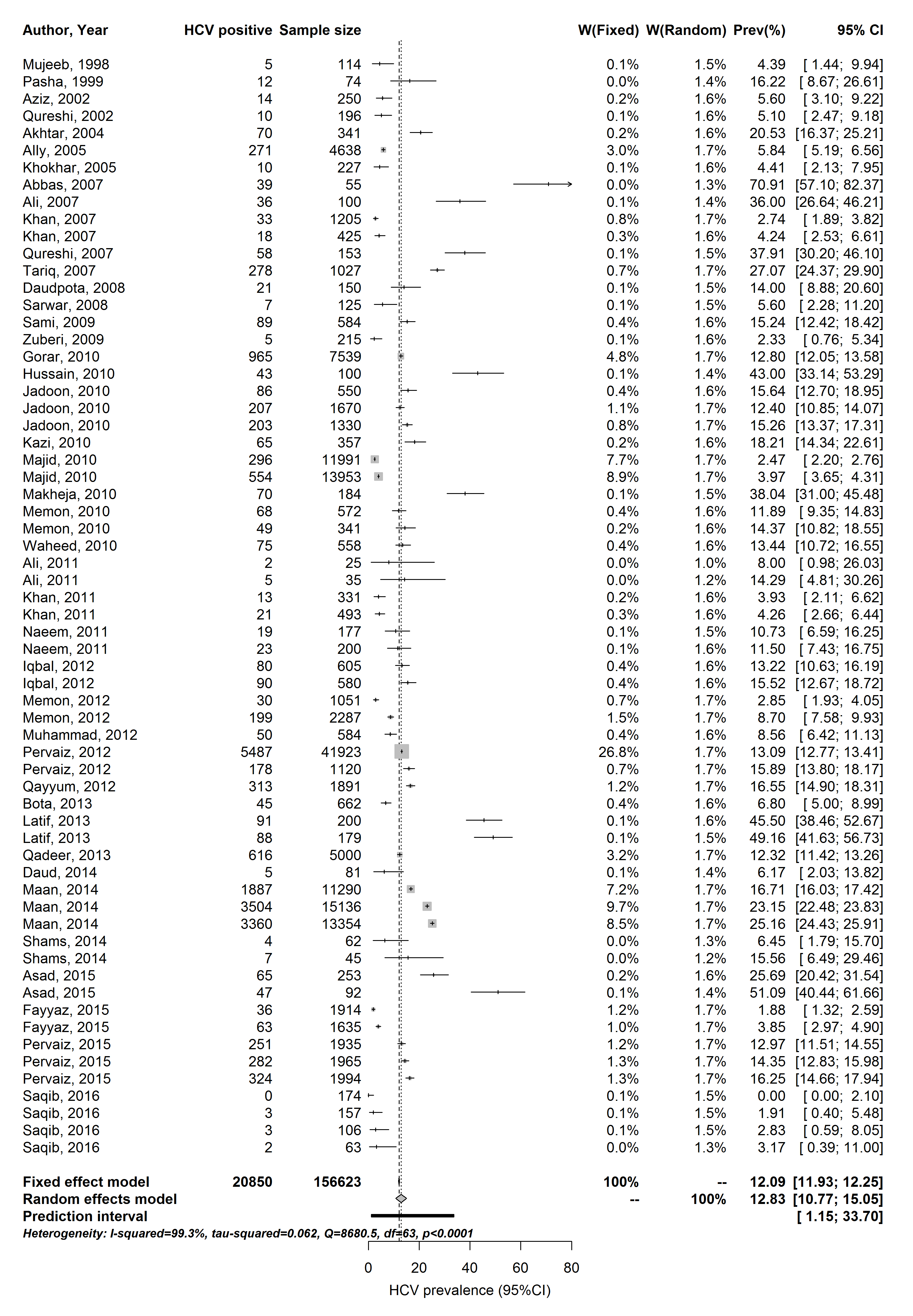
**

**Figure S5.** Forest plot of studies reporting hepatitis C virus (HCV) prevalence among special clinical populations in Pakistan.

**
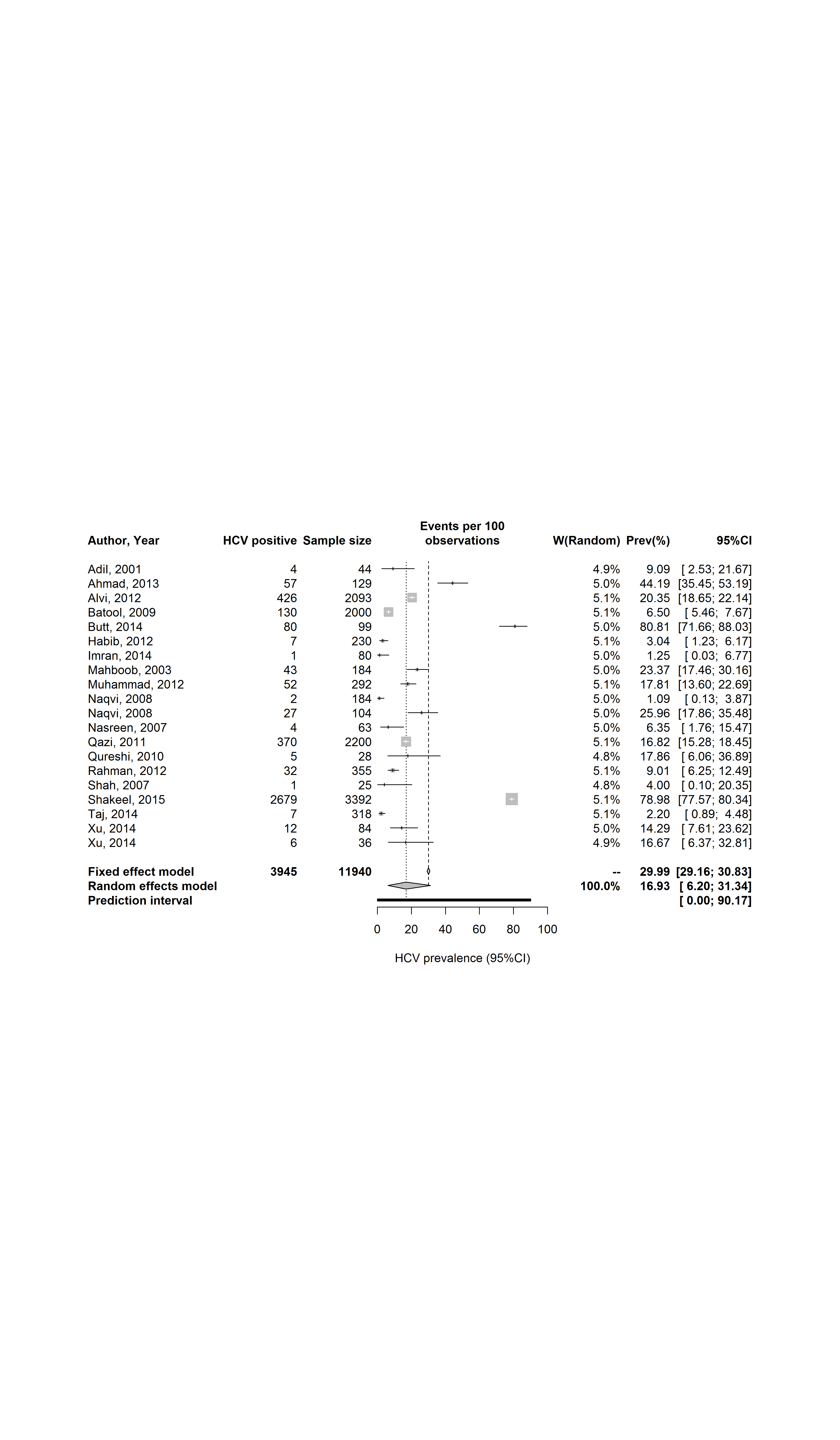
**

**Figure S6.** Forest plot of studies reporting hepatitis C virus (HCV) prevalence among populations with liver-related conditions in Pakistan.

**
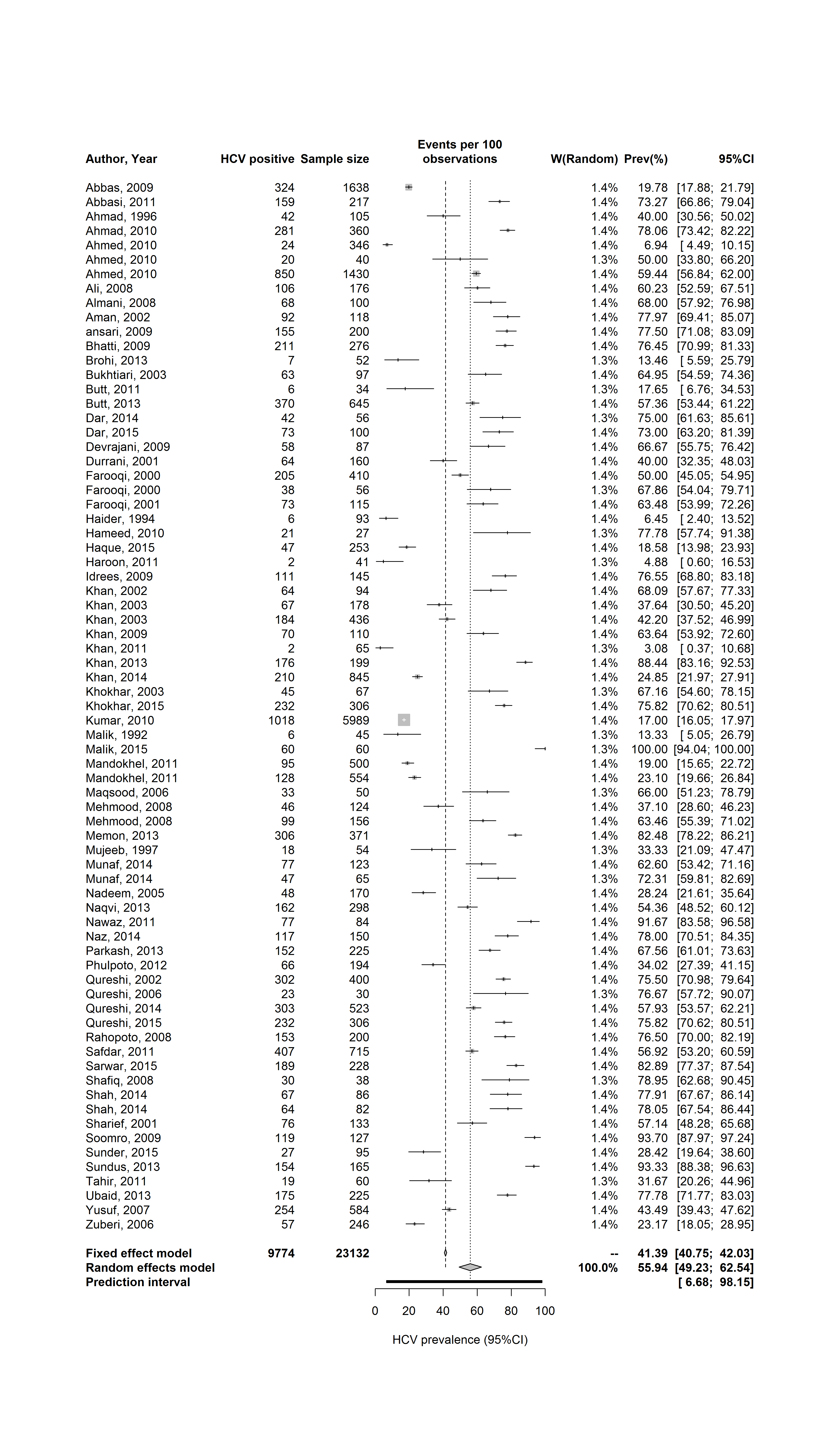
**

**Figure S7.** Forest plot of studies reporting hepatitis C virus (HCV) prevalence among people who inject drugs (PWID) in Pakistan.

**
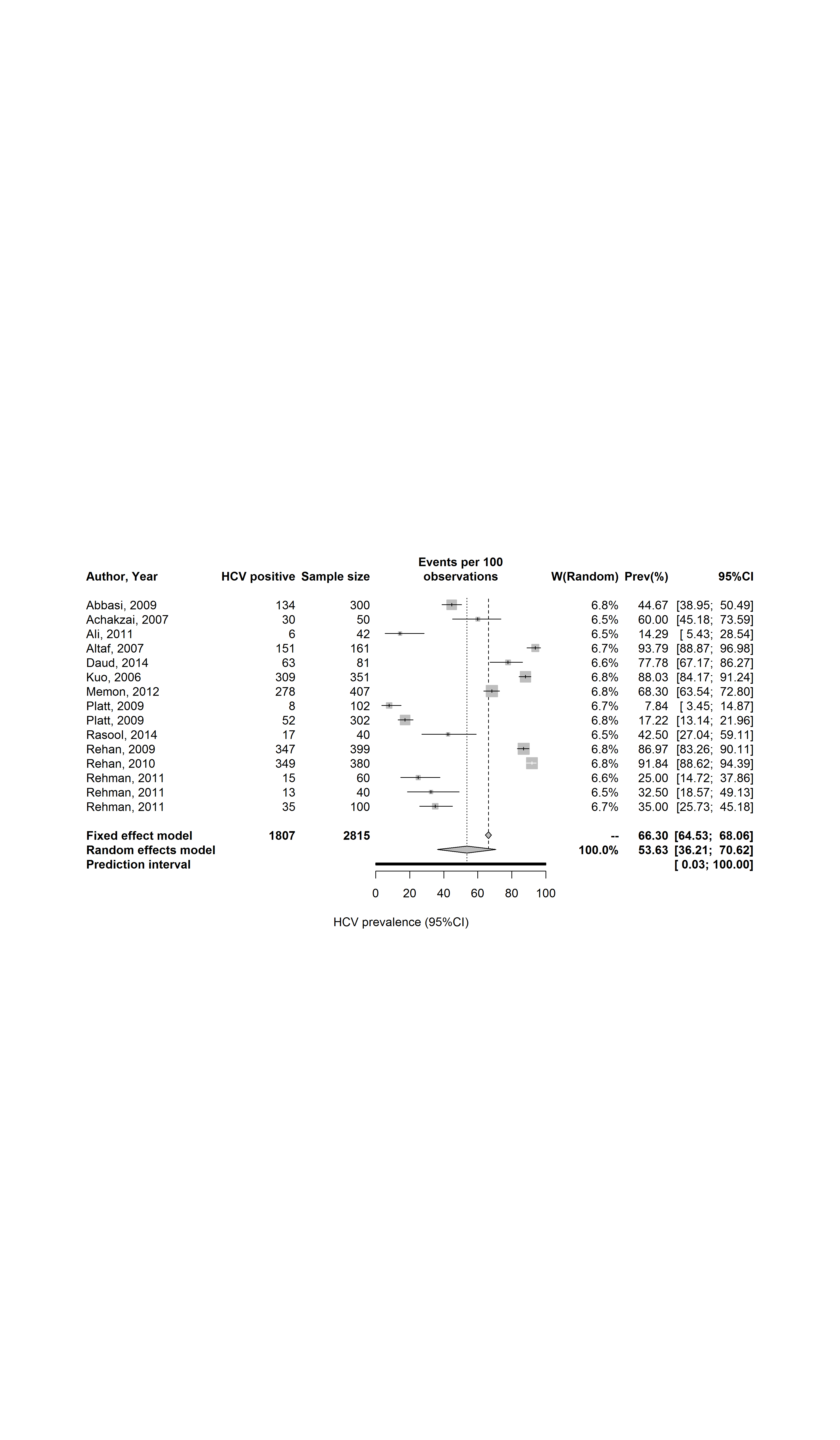
**

**Table S6.** Studies reporting hepatitis C virus (HCV) ribonucleic acid (RNA) prevalence among HCV antibody positive individuals (that is HCV viremic rate) in Pakistan.

| Author, year (citation) | Year(s) of data collection | Population’s classification based on risk of HCV exposure | Population | Number of HCV Ab positive individuals tested for RNA | HCV viremic rate (%) |
| --- | --- | --- | --- | --- | --- |
| Aziz, 2011 [120] | 2005-09 | Low risk | Pregnant woman | 510 | 79.7 |
| Donchuk, 2016 [121] | 2015-16 | Low risk | Outpatients | 998 | 90.2 |
| Idrees, 2008 [122] | 1999-07 | Low risk | General population | 422 | 49.2 |
| Idrees, 2008 [122] | 1999-07 | Low risk | General population | 71 | 50.4 |
| Karim, 2016 [123] | 2015 | Low risk | Blood donors | 56 | 93.3 |
| Khokhar, 2004 [124] | 2001-02 | Low risk | Pregnant woman | 13 | 72.2 |
| Mahboob, 2003 [43] | 1999-01 | Special clinical population | Lichen planus patients | 10 | 62.5 |
| Qureshi, 2007 [12] | NS | Intermediate risk | Spouses of index patients | 16 | 76.2 |
| Rauf, 2011 [125] | 2009 | Low risk | Refugees | 17 | 50.0 |
| Rauf, 2011 [125] | 2009 | Low risk | Refugees | 8 | 44.4 |
| Sundus, 2013 [126] | 2009-10 | Populations with liver conditions | Hepatitis patients | 148 | 98.0 |
| Zuberi, 2009 [17] | 2004-08 | Intermediate risk | Healthcare workers | 9 | 90.0 |

**References**

1. Moher D, Liberati A, Tetzlaff J, Altman DG, The PG. Preferred Reporting Items for Systematic Reviews and Meta-Analyses: The PRISMA Statement. PLoS Med. 2009;6.

2. Mujeeb S, Khatri Y, Khanani R. Frequency of parenteral exposure and seroprevalence of HBV, HCV, and HIV among operation room personnel. Journal of Hospital Infection. 1998;38(2):133-7.

3. Pasha O, Luby SP, Khan AJ, Shah SA, McCormick JB, Fisher-Hoch SP. Household members of hepatitis C virus-infected people in Hafizabad, Pakistan: Infection by injections from health care providers. Epidemiology and Infection. 1999;123(3):515-8. doi: <http://dx.doi.org/10.1017/S0950268899002770>. PubMed PMID: 30076936.

4. Aziz S, Memon A, Tily HI, Rasheed K, Jehangir K, Quraishy MS. Prevalence of HIV, hepatitis B and C amongst health workers of Civil Hospital Karachi. Jpma. 2002;The Journal of the Pakistan Medical Association. 52(3):92-4. PubMed PMID: 35607176.

5. Qureshi H, Ahsan T, Mujeeb SA, Jawad F, Mehdi I, Ahmed W, et al. Diabetes mellitus is equally frequent in chronic HCV and HBV infection. Jpma. 2002;The Journal of the Pakistan Medical Association. 52(7):280-3. PubMed PMID: 35540447.

6. Akhtar S, Moatter T. Intra-household clustering of hepatitis C virus infection in Karachi, Pakistan. Transactions of the Royal Society of Tropical Medicine and Hygiene. 2004;98(9):535-9. doi: <http://dx.doi.org/10.1016/j.trstmh.2003.12.010>. PubMed PMID: 38987089.

7. Ally SH, Ahmed A, Hanif R. An audit of serological tests carried out at clinical laboratory of Ayub Teaching Hospital, Abbottabad. Journal of Ayub Medical College, Abbottabad : JAMC. 2005;17(2):75-8. PubMed PMID: 41484771.

8. Khokhar N, Gill ML, Yawar A. Interspousal transmission of hepatitis C virus. Journal of the College of Physicians and Surgeons Pakistan. 2005;15(10):587-9. PubMed PMID: 43173622.

9. Abbas SZ, Batool SA, Pathan I, Muhammad SR, Abbas SQ. Liver diseases: Admissions and mortality in a medical ICU at a rural centre in Pakistan. Pakistan Journal of Medical Sciences. 2007;23(5):713-6. PubMed PMID: 350242054.

10. Ali SS, Ali IS, Aamir AH, Jadoon Z, Inayatullah S. Frequency of hepatitis C infection in diabetic patients. Journal of Ayub Medical College, Abbottabad : JAMC. 2007;19(1):46-9. PubMed PMID: 350078494.

11. Khan MS, Jamil M, Jan S, Zardad S, Sultan S, Sahibzada AS. Prevalence of hepatitis 'B' and 'C' in orthopaedics patients at Ayub Teaching Hospital Abbottabad. Journal of Ayub Medical College, Abbottabad : JAMC. 2007;19(4):82-4. PubMed PMID: 352473718.

12. Qureshi H, Arif A, Ahmed W, Alam SE. HCV exposure in spouses of the index cases. JPMA The Journal of the Pakistan Medical Association. 2007;57(4):175-7. PubMed PMID: 17489523.

13. Tariq FM, Chaudhry AA, Subhani H, Ul Haq I. Prevalence of hepatitis-C in Faisalabad region of Pakistan. Medical Forum Monthly. 2007;18(9):18-20. PubMed PMID: 351310046.

14. Daudpota AQ, Soomro AW. Sero prevalence of hepatitis B and C in surgical patients. Pakistan Journal of Medical Sciences. 2008;24(3):483-4. PubMed PMID: 351947453.

15. Sarwar J, Gul N, Idris M, Anis ur R, Farid J, Adeel MY. Seroprevalence of hepatitis B and hepatitis C in health care workers in Abbottabad. Journal of Ayub Medical College, Abbottabad : JAMC. 2008;20(3):27-9. PubMed PMID: 604088419.

16. Sami S, Korejo R, Bhutta SZ. Prevalence of hepatitis B and C: A Jinnah Postgraduate Medical Centre experience. Journal of Obstetrics and Gynaecology Research. 2009;35(3):533-8. doi: <http://dx.doi.org/10.1111/j.1447-0756.2008.00991.x>. PubMed PMID: 354905885.

17. Zuberi BF, Zuberi FF, Hasan SR, Kumar R, Memon SA, Afsar S. Frequency of acute hepatitis C after needle stick injury and its treatment outcome. Pakistan Journal of Medical Sciences. 2009;25(5):766-9. PubMed PMID: 355540598.

18. Gorar ZA, Zulfikar I. Seropositivity of hepatitis C in prison inmates of Pakistan - A cross sectional study in prisons of Sindh. Journal of the Pakistan Medical Association. 2010;60(6):476-9. PubMed PMID: 358913836.

19. Hussain T, Sosorburam T, Samdan A, Hayat A, Shu LY, Deo P. Concurrent hepatitis C and diabetes mellitus; An upcoming challenge in South East Asia. International Journal of Infectious Diseases. 2010;14:S73. doi: <http://dx.doi.org/10.1016/S1201-9712%2810%2960224-X>. PubMed PMID: 70216104.

20. Jadoon N, Shahzad A, Yaqoob R. Frequency of hepatitis C virus infection in Pakistani patients with type 2 diabetes mellitus. International Journal of Infectious Diseases. 2010;14:S68. doi: <http://dx.doi.org/10.1016/S1201-9712%2810%2960208-1>. PubMed PMID: 70216088.

21. Jadoon NA, Shahzad MA, Yaqoob R, Hussain M, Ali N. Seroprevalence of hepatitis C in type 2 diabetes: Evidence for a positive association. Virology Journal. 2010;7 (no pagination)(304). doi: <http://dx.doi.org/10.1186/1743-422X-7-304>. PubMed PMID: 51142846.

22. Kazi AM, Shah SA, Jenkins CA, Shepherd BE, Vermund SH. Risk factors and prevalence of tuberculosis, human immunodeficiency virus, syphilis, hepatitis B virus, and hepatitis C virus among prisoners in Pakistan. International Journal of Infectious Diseases. 2010;14(SUPPL. 3):e60-e6. doi: <http://dx.doi.org/10.1016/j.ijid.2009.11.012>. PubMed PMID: 50810236.

23. Majid A, Khan MS, Ullah S. Rising prevalence of hepatitis B and C and risk factors at District Headquarter Teaching Hospital Bannu, Khyber-Pakhtunkhwa. Journal of the College of Physicians and Surgeons Pakistan. 2010;20(7):492-3. PubMed PMID: 359397988.

24. Makheja KD, Abro AH, Kumar S. Sero-prevalence of hepatitis c antibodies in the people visiting roadside barbers. Pakistan Journal of Medical Sciences. 2010;26(2):402-6. PubMed PMID: 359164644.

25. Memon MR, Shaikh AA, Soomro AA, Arshad S, Shah QA. Frequency of hepatitis B and C in patients undergoing elective surgery. Journal of Ayub Medical College, Abbottabad : JAMC. 2010;22(2):167-70. PubMed PMID: 362241485.

26. Khan S, Attaullah S, Ayaz S, Niaz Khan S, Shams S, Ali I, et al. Molecular epidemiology of HCV among health care workers of Khyber Pakhtunkhwa. Virology Journal. 2011;8 (no pagination)(105). doi: <http://dx.doi.org/10.1186/1743-422X-8-105>. PubMed PMID: 51317660.

27. Naeem SS, Siddiqui EU, Kazi AN, Khan S, Abdullah FE, Adhi I. Prevalence of hepatitis 'B' and hepatitis 'C' among preoperative cataract patients in Karachi. BMC research notes. 2012;5:492. PubMed PMID: 366377635.

28. Iqbal MR, Rafi Y, Islam HRU, Abid KJ. Screening for hepatitis B and C in a surgical ward. Pakistan Journal of Medical and Health Sciences. 2012;6(3):564-7. PubMed PMID: 365901275.

29. Memon AR, Shafique K, Memon A, Draz AU, Rauf MUA, Afsar S. Hepatitis B and C prevalence among the high risk groups of Pakistani population. A cross sectional study. Archives of Public Health. 2012;70(1):9-. doi: 10.1186/0778-7367-70-9. PubMed PMID: PMC3502333.

30. Muhammad SK, Chandio MA, Soomro MA, Shaikh BA. Hepatitis C virus infection in non-Hodgkin's lymphoma: A case-control study. Hepatitis Monthly. 2012;12(1):16-22. doi: <http://dx.doi.org/10.5812/kowsar.1735143X.801>. PubMed PMID: 364217373.

31. Pervaiz A, Ghafoor T, Asghar RJ. Screening of prisoners for Human Immunodeficiency Virus (HIV), Hepatitis C (HCV) and B (HBV) in Punjab Province, Pakistan, 2009. International Journal of Infectious Diseases. 2012;16:e359. doi: <http://dx.doi.org/10.1016/j.ijid.2012.05.449>. PubMed PMID: 70869302.

32. Qayyum FA, Mahmood K, Siraj MR. Prevalence of blood borne diseases (hepatitis B & C) and strategy to protect health care workers. Pakistan Journal of Medical and Health Sciences. 2012;6(3):640-3. PubMed PMID: 365901297.

33. Bota R, Ahmed M. Frequency of hepatitis B and C in surgical patients, Civil Hospital Karachi. Hepatology International. 2013;7:S162. doi: <http://dx.doi.org/10.1007/s12072-013-9429-0>. PubMed PMID: 71307887.

34. Latif MZ, Hussain I, Nizami R, Dar U. Prevalence of hepatitis B & C in patients visiting a free eye camp for cataract surgery at Jarranwala District Faisalabad. Pakistan Journal of Medical and Health Sciences. 2013;7(2). PubMed PMID: 370001589.

35. Qadeer MI, Hasnain S, Yasmeen H. Sero-prevalence of sexually transmitted disease (Hiv, Syphilis, Hepatitis-B and Hepatitis-C) in Volunteer Donors of Gaol Inmates and Student Community in Punjab Province of Pakistan. Sexually Transmitted Infections Conference: STI and AIDS World Congress. 2013;89(no pagination). doi: <http://dx.doi.org/10.1136/sextrans-2013-051184.0820>. PubMed PMID: 71442852.

36. Maan MA, Fatma H, Muhammad J. Epidemiology of hepatitis C viral infection in Faisalabad, Pakistan: A retrospective study (2010-2012). African Health Sciences. 2014;14(4):810-5. doi: <http://dx.doi.org/10.4314/ahs.v14i4.6>. PubMed PMID: 601735338.

37. Shams N, Usmani F, Kumar N, Motwani R, Dahhri MM, Shaikh Z. Newly diagnosed hepatitis-B and hepatitis-C during surgical pre-operative assessment of patients from lower socioeconomic class; frequency, risk factors and vaccination status. Journal of the Liaquat University of Medical and Health Sciences. 2014;13(3):106-11. PubMed PMID: 605161690.

38. Asad M, Ahmed F, Zafar H, Farman S. Frequency and determinants of hepatitis B and C virus in general population of Farash Town, Islamabad. Pakistan Journal of Medical Sciences. 2015;31(6):1394-8. doi: <http://dx.doi.org/10.12669/pjms.316.7047>. PubMed PMID: 607142355.

39. Fayyaz M, Ghous SM, Fahimullah, Abbas I, Ahmed N, Ahmed A. Frequency of Hepatitis B and C in Patients Seeking Treatment at the Dental Section of a Tertiary Care Hospital. Journal of Ayub Medical College, Abbottabad : JAMC. 2015;27(2):395-7. PubMed PMID: 606608985.

40. Pervaiz A, Sipra FS, Rana TH, Qadeer I. Pre-Donation Screening of Volunteer Prisoner Blood Donors for Hepatitis B and C in Prisons of Punjab, Pakistan. Journal of Ayub Medical College, Abbottabad : JAMC. 2015;27(4):794-7. PubMed PMID: 611473148.

41. Saqib S, Khan MZ, Gardyzi SIHS, Qazi J. Prevalence and epidemiology of blood borne pathogens in health care workers of Rawalpindi/Islamabad. Journal of the Pakistan Medical Association. 2016;66(2):170-3. PubMed PMID: 607824736.

42. Adil SN, Burney IA, Kakepoto GN, Khurshid M. Epidemiological features of aplastic anaemia in Pakistan. Jpma. 2001;The Journal of the Pakistan Medical Association. 51(12):443-5. PubMed PMID: 35588489.

43. Mahboob A, Haroon TS, Iqbal Z, Iqbal F, Butt AK. Frequency of anti-HCV antibodies in patients with lichen planus. Journal of the College of Physicians and Surgeons Pakistan. 2003;13(5):248-51. PubMed PMID: 36763852.

44. Nasreen S, Ahmed I, Wahid Z. Associations of lichen planus: A study of 63 cases. Journal of Pakistan Association of Dermatologists. 2007;17(1):17-20. PubMed PMID: 47083787.

45. Naqvi SAA, Rizvi SAH, Zafar MN, Ahmed E, Ali B, Mehmood K, et al. Health status and renal function evaluation of kidney vendors: A report from Pakistan. American Journal of Transplantation. 2008;8(7):1444-50. doi: <http://dx.doi.org/10.1111/j.1600-6143.2008.02265.x>. PubMed PMID: 351994125.

46. Batool A, Khan MI, Bano KA. Efficacy of immunoassay chromatography test for hepatitis-C antibodies detection. Journal of Ayub Medical College, Abbottabad : JAMC. 2009;21(3):38-9. PubMed PMID: 359877867.

47. Waheed A, Zaeem FA, Shariff MM, Qayyum A. Operating in a yellow nation; the frequency of hepatitis B and hepatitis C positive at a tertiary care teaching hospital. JPMA The Journal of the Pakistan Medical Association. 2010;60(12):1058-60.

48. Qazi MA, Raza H, Imran A, Anjum S, Chaudhary GMUD. Comparison of risk factors for the spread of chronic HBV and chronic HCV infection (a local experience: Southern Punjab, Pakistan). Hepatology International. 2011;5 (1):236. doi: <http://dx.doi.org/10.1007/s12072-010-9241-z>. PubMed PMID: 70369623.

49. Alvi H, Qureshi A. Frequency of hepatitis B and C in patients reported at Al-Tibri Medical College & Hospital. Medical Forum Monthly. 2012;23(11):11-3. PubMed PMID: 368163221.

50. Habib A, Raza N. Clinical pattern of vitiligo. Journal of the College of Physicians and Surgeons Pakistan. 2012;22(1):61-2. PubMed PMID: 365335506.

51. Rahman A, Rizvi SD, Sheikh ZI. Frequency of HCV infection in different dermatological disorders. Journal of Ayub Medical College, Abbottabad : JAMC. 2012;24(2):58-61. PubMed PMID: 372447817.

52. Ahmad Z, Arshad H, Idrees R, Ud-Din N, Ahmed R, Ahmed A, et al. Gastrointestinal, liver and biliary tract pathology: A histopathological and epidemiological perspective from Pakistan with a review of the literature. Asian Pacific Journal of Cancer Prevention. 2013;14(11):6997-7005. doi: <http://dx.doi.org/10.7314/APJCP.2013.14.11.6997>. PubMed PMID: 1372122925.

53. Butt N, Butt S, Rani T, Abbasi A. Esophageal variceal band ligation experience in patients with portal hypertension: a single centre study. Journal of Gastroenterology and Hepatology. 2014;29:50.

54. Imran M, Adnan M, Ali Z. Histopathologic and clinicopathologic correlations in children with atypical nephrotic syndrome. Medical Forum Monthly. 2014;25(4):23-7. PubMed PMID: 373680861.

55. Taj M, Shamsi TS, Ansari SH, Farzana T, Nazi A, Nadeem M, et al. Epidemiologic and HLA antigen profile in patients with aplastic anemia. Journal of the College of Physicians and Surgeons--Pakistan : JCPSP. 2014;24(8):549-52. doi: 08.2014/jcpsp.549552. PubMed PMID: 25149831.

56. Xu X, Sajid KM, Zhai X, Yao W, Mir S, Mahmood R, et al. Prevalence of hepatitis B and C virus in euthyroid patients. Tropical Journal of Pharmaceutical Research. 2014;13(10):1703-6. doi: <http://dx.doi.org/10.4314/tjpr.v13i10.19>. PubMed PMID: 600313537.

57. Shakeel S, Mehmood A, Gul A, Hameed A, Tayyab GUN. Esophageal varices due to hepatitis C virus the most frequent endoscopic finding in upper gastrointestinal bleed in Pakistani population. Hepatology International. 2015;1):S273-S4. doi: <http://dx.doi.org/10.1007/s12072-015-9609-1>. PubMed PMID: 71806879.

58. Khan S, Rai MA, Khan A, Farooqui A, Kazmi SU, Ali SH. Prevalence of HCV and HIV infections in 2005-earthquake-affected areas of Pakistan. BMC Infectious Diseases. 2008;8 (no pagination)(147). doi: <http://dx.doi.org/10.1186/1471-2334-8-147>. PubMed PMID: 352727580.

59. Abdullah FE, Pasha HA, Memon AA, Shah UN. Increasing frequency of anti-HCV seropositivity in a cross-section of people in Karachi, Pakistan. Pakistan Journal of Medical Sciences. 2011;27(4):767-70. PubMed PMID: 362600646.

60. Ali I, Siddique L, Rehman LU, Khan NU, Iqbal A, Munir I, et al. Prevalence of HCV among the high risk groups in Khyber Pakhtunkhwa. Virology Journal. 2011;8 (no pagination)(296). doi: <http://dx.doi.org/10.1186/1743-422X-8-296>. PubMed PMID: 51474733.

61. Haider Z, Khan AA, Rehman K, Janjua MI, Iqbal J, Chishti MA, et al. Sero-diagnosis for viral hepatitis in 93 patients admitted with acute hepatitis in three different teaching hospitals in Lahore. Jpma. 1994;The Journal of the Pakistan Medical Association. 44(8):182-4. PubMed PMID: 125005605.

62. Tong CYW, Khan R, Beeching NJ, Tariq WUZ, Hart CA, Ahmad N, et al. The occurrence of hepatitis B and C viruses in Pakistani patients with chronic liver disease and hepatocellular carcinoma. Epidemiology and Infection. 1996;117(2):327-32. PubMed PMID: 26345242.

63. Mujeeb SA, Jamal Q, Khanani R, Iqbal N, Kaher S. Prevalence of hepatitis B surface antigen and HCV antibodies in hepatocellular carcinoma cases in Karachi, Pakistan. Tropical Doctor. 1997;27(1):45-6. PubMed PMID: 27064371.

64. Farooqi JI, Farooqi RJ. Relative frequency of Hepatitis 'B' virus and Hepatitis 'C' virus infections in patients of cirrhosis in NWFP. Journal of the College of Physicians and Surgeons Pakistan. 2000;10(6):217-9. PubMed PMID: 30490177.

65. Farooqi JI, Farooqi RJ. Relative frequency of Hepatitis 'B' and 'C' virus infections in cases of Hepatocellular Carcinoma in North-West Frontier Province, Pakistan. Journal of the College of Physicians and Surgeons Pakistan. 2000;10(4):128-30. PubMed PMID: 30333562.

66. Durrani AB, Rana AB, Siddiqi HS, Marwat BU. The spectrum of chronic liver disease in Balochistan. Medical Forum Monthly. 2001;12(4):20-2. PubMed PMID: 34097973.

67. Farooqi JI, Farooqi RJ. Predictors of the outcome after the first episode of acute variceal bleeding in liver cirrhosis patients. Journal of the College of Physicians and Surgeons Pakistan. 2001;11(6):379-82. PubMed PMID: 32685140.

68. Sharieff S, Burney IA, Ahmad N, Salam A, Siddiqui T. Radiological features of hepatocellular carcinoma in Southern Pakistan. Tropical Doctor. 2001;31(4):224-5. PubMed PMID: 32980365.

69. Aman-ur-Rehman A, Murad S. Hepatocellular carcinoma: A retrospective analysis of 118 cases. Journal of the College of Physicians and Surgeons Pakistan. 2002;12(2):108-9. PubMed PMID: 34257608.

70. Khan AA, Khalil-ur-Rehman A, Haider Z, Shafqat F. Seromarkers of hepatitis B and C in patients with cirrhosis. Journal of the College of Physicians and Surgeons Pakistan. 2002;12(2):105-7. PubMed PMID: 34257607.

71. Bukhtiari N, Hussain T, Iqbal M, Malik AM, Qureshi AH, Hussain A. Hepatitis B and C single and co-infection in chronic liver disease and their effect on the disease pattern. Jpma. 2003;The Journal of the Pakistan Medical Association. 53(4):136-40. PubMed PMID: 36727548.

72. Khan TS, Rizvi F, Rashid A. Hepatitis C seropositivity among chronic liver disease patients in Hazara, Pakistan. Journal of Ayub Medical College, Abbottabad : JAMC. 2003;15(2):53-5. PubMed PMID: 37326262.

73. Khokhar N, Aijazi I, Gill ML. Spectrum of hepatocellular carcinoma at Shifa International Hospital, Islamabad. Journal of Ayub Medical College, Abbottabad : JAMC. 2003;15(4):1-4. PubMed PMID: 38563713.

74. Nadeem M, Yousaf MA, Zakaria M, Hussain T, Ali N. The value of clinical signs in diagnosis of cirrhosis. Pakistan Journal of Medical Sciences. 2005;21(2):121-4. PubMed PMID: 40978941.

75. Maqsood S, Saleem A, Iqbal A, Butt JA. Precipitating factors of hepatic encephalopathy: experience at Pakistan Institute of Medical Sciences Islamabad. Journal of Ayub Medical College, Abbottabad : JAMC. 2006;18(4):58-62. PubMed PMID: 47406006.

76. Zuberi BF, Quraishy MS, Afsar S, Kazi LAG, Memon AR, Qadeer R, et al. Frequency and comparative analysis of hepatitis D in patients seeking treatment for hepatitis B. Journal of the College of Physicians and Surgeons Pakistan. 2006;16(9):581-4. PubMed PMID: 44566685.

77. Yusuf MA, Badar F, Meerza F, Khokhar RA, Ali FA, Sarwar S, et al. Survival from hepatocellular carcinoma at a cancer hospital in Pakistan. Asian Pacific journal of cancer prevention : APJCP. 2007;8(2):272-4. PubMed PMID: 350344302.

78. Ali M, Abbas SZ, Sultana F, Akhtar W, Shaw S, Abbas SQ. Non-B, non-C hepatitis as a cause of advanced chronic liver disease requiring medical admission at a rural centre in Pakistan. Pakistan Journal of Medical Sciences. 2008;24(2):278-82. PubMed PMID: 351798613.

79. Almani SA, Memon AS, Memon AI, Shah MI, Rahpoto MQ, Solangi R. Cirrhosis of liver: Etiological factors, complications and prognosis. Journal of the Liaquat University of Medical and Health Sciences. 2008;7(2):61-6. PubMed PMID: 352341084.

80. Rahopoto MQ, Almani SA, Shaikh MA, Shah MI, Maheshwari N, Baloch GH, et al. Frequency of night blindness in cirrhosis and effective restoration of vision with vitamin A therapy. Journal of the Liaquat University of Medical and Health Sciences. 2008;7(2):75-8. PubMed PMID: 352341087.

81. Abbas SZ, Muhammad AH, Shaw S. Frequency of hepatitis C virus infection and its genotypes among patients attending a liver clinic and voluntary blood donors in a rural area of Pakistan. Hepatology International. 2009;3 (1):170. doi: <http://dx.doi.org/10.1007/s12072-009-9123-4>. PubMed PMID: 70060277.

82. Ansari S, Memon MS, Devrajani BR. Frequency of hepatitis B and hepatitis C in patients with hepatocellular carcinoma at hyderabad. Journal of the Liaquat University of Medical and Health Sciences. 2009;8(2):109-12. PubMed PMID: 358300207.

83. Bhatti SM, Aman-ur-Rehman A, Sohail A, Randhawa IA, Hyder SW. Hepatocellular carcinoma: A retrospective analysis of 276 cases. European Journal of Cancer, Supplement. 2009;7 (2-3):393. PubMed PMID: 70210749.

84. Devrajani BR, Shah SZA, Devrajani T, Kumar D. Precipitating factors of hepatic encephalopathy at a tertiary care hospital Jamshoro, Hyderabad. JPMA The Journal of the Pakistan Medical Association. 2009;59(10):683.

85. Idrees M, Rafique S, Rehman IU, Akbar H, Yousaf MZ, Butt S, et al. Hepatitis C virus genotype 3a infection and hepatocellular carcinoma: Pakistan experience. World Journal of Gastroenterology. 2009;15(40):5080-5. doi: <http://dx.doi.org/10.3748/wjg.15.5080>. PubMed PMID: 358052382.

86. Khan P, Ahmad A, Muhammad N, Khan TM, Ahmad B. Screening of 110 cirrhotic patients for hepatitis B and C at Saidu Teaching Hospital Saidu Sharif Swat. Journal of Ayub Medical College, Abbottabad : JAMC. 2009;21(1):119-21. PubMed PMID: 360305214.

87. Soomro AA, Devrajani BR, Shaikh K, Shah SZA, Devrajani T, Bibi I. Serum zinc level in patients with liver cirrhosis. Pakistan Journal of Medical Sciences. 2009;25(6):986-91. PubMed PMID: 358046989.

88. Ahmed S, Mumtaz K, Ahmed US, Shah HA, Abid S, Hamid S, et al. Frequency and characteristic features of portal hypertensive gastropathy in patients with viral cirrhosis. Journal of the College of Physicians and Surgeons Pakistan. 2010;20(11):714-8. PubMed PMID: 360039383.

89. Ahmed W, Qureshi H, Arif A, Alam SE. Changing trend of viral hepatitis - "A twenty one year report from Pakistan Medical Research Council Research Centre, Jinnah Postgraduate Medical Centre, Karachi". Journal of the Pakistan Medical Association. 2010;60(2):86-9. PubMed PMID: 358210442.

90. Kumar A, Lalani S, Afridi AA, Khuwaja AK. Screening of hepatitis B and C among people visiting general practice clinics in a rural district of Sindh, Pakistan. Journal of Ayub Medical College, Abbottabad : JAMC. 2010;22(4):143-5. PubMed PMID: 364642540.

91. Mehmood K, Muhammad N, Jan A. FREQUENCY OF HEPATITIS B AND C VIRAL MARKERS IN PATIENTS OF CIRRHOSIS LIVER IN THE NORTH WEST FRONTIER PROVINCE. Journal of Postgraduate Medical Institute (Peshawar-Pakistan). 2011;22(2).

92. Safdar RM. Establishment of a viral hepatitis surveillance system--Pakistan. Centers for Disease Control Prevention, 2009-2011. MMWR Morbidity and mortality weekly report. 2011;60(40):1385.

93. Abbasi A, Bhutto AR, Butt N, Munir S, Dhillo AK. Frequency of portal hypertensive gastropathy and its relationship with biochemical, haematological and endoscopic features in cirrhosis. J Coll Physicians Surg Pak. 2011;21(12):723-6.

94. Khan A, Tanaka Y, Kurbanov F, Elkady A, Abbas Z, Azam Z, et al. Investigating an outbreak of acute viral hepatitis caused by hepatitis E virus variants in Karachi, South Pakistan. Journal of medical virology. 2011;83(4):622-9.

95. Mandokhel SK, Saleem M, Khalid SR, Ullah A. Frequency of hepatitis c in patients of chronic liver disease in balochistan. Pakistan Journal of Medical and Health Sciences. 2011;5(2):280-2. PubMed PMID: 365655112.

96. Nawaz A, Chaudhry A, Riaz M, Yousaf M, Alvi A, Batool S, et al. Presentation of hepatocellular cancer in the developing world: An analysis of the presentation and association with chronic hepatitis B and C. American Journal of Gastroenterology. 2011;106:S126. doi: <http://dx.doi.org/10.1038/ajg.2011.336_5>. PubMed PMID: 70556107.

97. Tahir A, Malik FR, Ahmad I, Akhtar P. Aetiological factors of chronic liver disease in children. Journal of Ayub Medical College, Abbottabad : JAMC. 2011;23(2):12-4. PubMed PMID: 373389825.

98. Phulpoto JA, Shah IA, Bhatti Z. Prevalence of hepatocellular carcinoma in cirrhotic patients of Northern Sindh attending liver clinics at Ghulam Mohammad Mahar Medical College Hospitals Sukkur and Khairpur. Journal of the Liaquat University of Medical and Health Sciences. 2012;11(1):29-33. PubMed PMID: 365049278.

99. Brohi ZP, Sadaf A, Perveen U. Etiology, clinical features and outcome of fulminant hepatic failure in pregnancy. Age (years). 2013;29(8.404):17-42.

100. Butt AS, Hamid S, Wadalawala AA, Ghufran M, Javed AA, Farooq O, et al. Hepatocellular carcinoma in Native South Asian Pakistani population; trends, clinico-pathological characteristics & differences in viral marker negative & viral-hepatocellular carcinoma. BMC research notes. 2013;6:137. doi: <http://dx.doi.org/10.1186/1756-0500-6-137>. PubMed PMID: 603055745.

101. Khan H, Virk S, Umar M, Khaar HTB, Qureshi I, Khan J, et al. HCV related HCC in Pakistan. Hepatology International. 2013;7:S617-S8. doi: <http://dx.doi.org/10.1007/s12072-013-9429-0>. PubMed PMID: 71309156.

102. Memon S, Zaki M, Kumar P, Bawany A, Humera M. Characteristics of hepatocellular carcinoma in diabetic patients. Hepatology International. 2013;7:S611. doi: <http://dx.doi.org/10.1007/s12072-013-9429-0>. PubMed PMID: 71309137.

103. Naqvi IH, Mahmood K, Salekeen S, Akhter ST. Determining the frequency and severity of malnutrition and correlating it with the severity of liver cirrhosis. Turkish Journal of Gastroenterology. 2013;24(5):415-22. doi: <http://dx.doi.org/10.4318/tjg.2013.0637>. PubMed PMID: 372107117.

104. Parkash O, Alishah H, Mohyuddin GR, Ayub A, Jafri W, Hamid S. Electrophysiological changes in patients with liver cirrhosis in tertiary care hospital Karachi Pakistan. Hepatology International. 2013;7:S529. doi: <http://dx.doi.org/10.1007/s12072-013-9429-0>. PubMed PMID: 71308898.

105. Sundus A, Siddique O, Ibrahim MF, Abbasi Z, Aziz S. Hepatitis patients lost to follow-up at a liver centre in a tertiary care hospital of Karachi, Pakistan - A cross-sectional descriptive study. Journal of the Pakistan Medical Association. 2013;63(12):1566-70. PubMed PMID: 370361398.

106. Ubaid M, Mahmood K. MELD and MELD-Na scoring in the patients of chronic liver disease due to chronic hepatitis B and C. Hepatology International. 2013;7:S673-S4. doi: <http://dx.doi.org/10.1007/s12072-013-9429-0>. PubMed PMID: 71309314.

107. Dar FS, Dogar AW, Zia HH, Liaqat A, Pervaiz M, Khan NA, et al. Living donor liver transplantation in Pakistan: A first report. Liver Transplantation. 2014;20:S263. doi: <http://dx.doi.org/10.1002/lt.23901>. PubMed PMID: 71562694.

108. Khan J, Shafiq M, Mushtaq S, Ayaz S, Ullah R, Abdei-Salam NM, et al. Seropositivity and coinfection of hepatitis B and C among patients seeking hospital care in Islamabad, Pakistan. BioMed Research International. 2014;2014 (no pagination)(516859). doi: <http://dx.doi.org/10.1155/2014/516859>. PubMed PMID: 373505801.

109. Munaf A, Memon MS, Kumar P, Ahmed S, Kumar MB. Comparison of viral hepatitis-associated hepatocellular carcinoma due to HBV and HCV - cohort from liver clinics in Pakistan. Asian Pacific journal of cancer prevention : APJCP. 2014;15(18):7563-7. PubMed PMID: 604959164.

110. Naz F, Bawany A, Khahro AA. Sexual dysfunction in the patients with chronic liver disease. Hepatology International. 2014;1):S299. doi: <http://dx.doi.org/10.1007/s12072-014-9519-7>. PubMed PMID: 71384553.

111. Qureshi MO, Shafqat F, Dar FS, Salih M, Khokhar N. Renal failure in patients with end stage liver disease and its impact on clinical outcome. Journal of the College of Physicians and Surgeons Pakistan. 2014;24(9):628-31. PubMed PMID: 373843306.

112. Shah HA, Azam Z, Rauf J, Abid S, Hamid S, Jafri W, et al. Carvedilol vs. esophageal variceal band ligation in the primary prophylaxis of variceal hemorrhage: A multicentre randomized controlled trial. Journal of Hepatology. 2014;60(4):757-64. doi: <http://dx.doi.org/10.1016/j.jhep.2013.11.019>. PubMed PMID: 52942167.

113. Dar FS, Bhatti ABH, Zia H, Amin S, Shah NH, Salih M, et al. Outcome of 100 LDLT recipients: First report from Pakistan. Transplantation. 2015;1):291. doi: <http://dx.doi.org/10.1097/01.tp.0000469973.81769.3c>. PubMed PMID: 72118796.

114. Haque IU, Zafar S, Iqbal W, Hassan GU, Farooq M, Nasir B, et al. Does value of alpha fetoprotein matter in HCC? Pakistan Journal of Medical and Health Sciences. 2015;9(1):305-7. PubMed PMID: 603502084.

115. Khokhar N, Qureshi MO, Ahmad S, Ahmad A, Khan HH, Shafqat F, et al. Comparison of once a day rifaximin to twice a day dosage in the prevention of recurrence of hepatic encephalopathy in patients with chronic liver disease. Journal of Gastroenterology and Hepatology (Australia). 2015;30(9):1420-2. doi: <http://dx.doi.org/10.1111/jgh.12970>. PubMed PMID: 605379825.

116. Malik K, Batool F, Khan SA, ul Amir Z. Platelet count/splenic size ratio as a non-invasive parameter to predict the presence of esophageal varices in cirrhotics. Rawal Medical Journal. 2015;40(4):371-4. PubMed PMID: 606852370.

117. Qureshi MO, Shafqat F, Salih M, Khokhar N. Daily single dose rifaximin for prevention of hepatic encephalopathy in patients with chronic liver disease. Hepatology International. 2015;1):S325. doi: <http://dx.doi.org/10.1007/s12072-015-9609-1>. PubMed PMID: 71807036.

118. Sarwar S, Khan AA, Tarique S. Predicting prognosis in hepatocellular carcinoma: Comparison of staging systems in Pakistani cohort. Journal of the College of Physicians and Surgeons Pakistan. 2015;25(9):648-53. PubMed PMID: 605950891.

119. Sunder JRM. Quacks are quick regarding management of viral infections (HBV, HCV) mainly in rural (desert) areas of Pakistan. Journal of Viral Hepatitis. 2015;22:64. doi: <http://dx.doi.org/10.1111/jvh.82-12425>. PubMed PMID: 71956232.

120. Aziz S, Hossain N, Karim SA, Rajper J, Soomro N, Noorulain W, et al. Vertical transmission of hepatitis C virus in low to middle socio-economic pregnant population of Karachi. Hepatology International. 2011;5(2):677-80. doi: <http://dx.doi.org/10.1007/s12072-010-9229-8>. PubMed PMID: 51212567.

121. Donchuk D, Rossi G, Bjorklund Y, Zainal HM, Auat R, Mazzeo V. Hepatitis C treatment in a primary care clinic in the high HCV burden setting in Karachi, Pakistan. Hepatology International. 2016;1):S34. doi: <http://dx.doi.org/10.1007/s12072-016-9707-8>. PubMed PMID: 72199986.

122. Idrees M, Lal A, Naseem M, Khalid M. High prevalence of hepatitis C virus infection in the largest province of Pakistan. Journal of Digestive Diseases. 2008;9(2):95-103. doi: <http://dx.doi.org/10.1111/j.1751-2980.2008.00329.x>. PubMed PMID: 351566631.

123. Karim F, Nasar A, Alam I, Alam I, Hassam S, Gul R. Incidence of active HCV infection amongst blood donors of Mardan District, Pakistan. Asian Pac J Cancer Prev. 2016;17:235-8.

124. Khokhar N, Raja KS, Javaid S. Seroprevalence of Hepatitis C Virus Infection and its Risk Factors in Pregnant Women. Journal of the Pakistan Medical Association. 2004;54(3):135. PubMed PMID: 38525221.

125. Rauf A, Nadeem MS, Ali A, Iqbal M, Mustafa M, Latif MM, et al. Prevalence of hepatitis B and C in internally displaced persons of war against terrorism in Swat, Pakistan. European journal of public health. 2011;21(5):638-42. PubMed PMID: 560025834.

126. Sundus A, Siddique O, Ibrahim MF, Abbasi Z, Aziz S. Hepatitis patients lost to follow-up at a liver centre in a tertiary care hospital of Karachi, Pakistan--a cross-sectional descriptive study. JPMA The Journal of the Pakistan Medical Association. 2013;63(12):1566-70.
